# Achieving of High Density/Utilization of Active Groups via Synergic Integration of C=N and C=O Bonds for Ultra-Stable and High-Rate Lithium-Ion Batteries

**DOI:** 10.1155/2018/1936735

**Published:** 2018-12-16

**Authors:** Tao Sun, Zong-Jun Li, Xin-Bo Zhang

**Affiliations:** ^1^State Key Laboratory of Rare Earth Resource Utilization, Changchun Institute of Applied Chemistry, Chinese Academy of Sciences, Changchun, 130022, China; ^2^University of Chinese Academy of Sciences, Beijing, 100049, China; ^3^State Key Laboratory of Electroanalytical Chemistry, Changchun Institute of Applied Chemistry, Chinese Academy of Sciences, Changchun, 130022, China

## Abstract

Organic electrode materials are receiving ever-increasing research interest due to their combined advantages, including resource renewability, low cost, and environmental friendliness. However, their practical applications are still terribly plagued by low conductivity, poor rate capability, solubility in electrolyte, and low density/utilization of active groups. In response, herein, as a proof-of-concept experiment, C=N and C=O bonds are synergically integrated into the backbone of pentacene to finely tune the electronic structures of pentacene. Unexpectedly, the firstly obtained unique 5,7,11,14-tetraaza-6,13-pentacenequinone/reduced graphene oxide (TAPQ/RGO) composite exhibits superior electrochemical performances, including an ultra-stable cycling stability (up to 2400 cycles) and good rate capability (174 mAh g^−1^ even at a high current density of 3.2 A g^−1^), which might be attributed to the abundant active groups, *π*-conjugated molecular structure, leaf-like morphology, and the interaction between TAPQ and graphene.

Lithium-ion batteries (LIBs) are generally proposed to meet the ever-increasing requirements of electrochemical energy storage for portable electronics and electrical vehicles; however, there have been great concerns on the costs correlated with mineral exploitation for LIBs manufacturing, which still heavily rely on unsustainable and unbiodegradable inorganic compounds [1–5]. In sharp contrast to inorganic electrode materials, organic compounds, characterized by abundant resources, structural diversity, and designability provide an alternative for developing sustainable energy materials and devices [6–11]. However, in the ocean of organic compounds, how to discover the potential high performance organic electrode material is still a daunting challenge [12–18]. Theoretically, taking full advantage of molecular design and deepen the understanding of the relationship between structure and property will speed up the research process on the exploration of advanced organic electrode material. However, up to now, how to realize the controllable fabrication from molecular design to excellent battery performances and even obtain practical application in full cell are still of paramount challenge.

As an important family of organic molecule, pentacene has been considered as a benchmark molecular in organic field effect transistors [19–22]. Theoretically, the introduction of different functional group into the planar skeleton of pentacene will endow its derivatives with unexpected properties and thus functionalized pentacene might be employed as important building blocks for organic electrode materials [23–25]. Especially, when the electroactive sites are introduced into the *π*-conjugated pentacene backbone, the resultant* N*-heteropentacenequinones will be the potential electrode material. However, in fact, to obtain a satisfactory electrochemical performance, several challenges need to be conquered—what kind of functional group should be chosen and how many active sites can be integrated. More seriously, like other organic molecular materials, the intrinsic low conductivity and dissolution problem in electrolyte will hinder its cycling stability and rate capability [26–30]. Up to now, there is no report on* N*-heteropentacenequinones as an exemplification to display the molecular design of organic electrode material, to say nothing with good cycling stability and rate capability.

Herein, as a proof-of-concept experiment, to systematically and finely tune the electronic structure of pentacene derivatives, C=N and C=O bonds are synergically inserted into the backbone of pentacene to achieve a cooperative effect and exert the optimum performances, which is then further integrated with reduced graphene oxide (RGO) to provide an additional energy barrier against the intrinsic dissolution of the pentacene-derivatives in electrolyte. Unexpectedly, the resultant novel 5,7,11,14-tetraaza-6,13-pentacenequinone/RGO (TAPQ/RGO) composite shows superior electrochemical performances, including an ultrastable cyclability (193 mAh g^−1^ after 2400 cycles at current density of 500 mA g^−1^) and rate capability (174 mAh g^−1^ at current density of 3200 mA g^−1^), which is rarely reported for organic small molecular electrode materials. More importantly, the stable full cell holds superior electrochemical performance (92% capacity retention after 220 cycles), possessing potential appliance value of TAPQ/RGO.

To develop high performance electrode material, the design of organic molecular should take full consideration of high redox potential and specific capacity. In the pursuit of high working potential, the introduction of electron-withdrawing group can efficiently lower the LUMO energies. On the other hand, bridging the carbonyl units with alkyl chains is a common method to obtain high specific capacity [7]. However, the inevitable introduction of inactive groups will increase the molecular weight and reduce the overall energy densities. In addition, each strategy can only meet one aspect of the requirements of high-performance electrode material. Therefore, a functional integration design strategy should avoid these disadvantages and try to meet the requirements for high-performance electrode material as much as possible, thus to achieve the high utilization of functional groups.

As a platform molecular, pentacene with an extended *π*-conjugated system can efficiently disperse negative charge by delocalization. Therefore, the systematic incorporation of C=N and C=O bonds into the backbone of pentacene might provide an exemplification to realize the above design idea. Besides being the active center to bound with lithium ion, the functions of C=N bond are far from being explored [14–17]. Under the premise of ensuring conjugation, replacing the aromatic carbon with sp^2^ hybridization nitrogen atom will increase the redox potential with minimal change of the molecular weight (a tiny increase of 2 g mol^−1^). In contrast, the incorporation of C=O bond will result in extra increase of molecular weight of 16 g mol^−1^. Therefore, it is more economical to introduce more C=N bond into the conjugated skeleton to form heteroaromatic system. However, it does not mean the unimportance of C=O bond. On the one hand, to achieve a high capacity, introducing more functional groups into conjugation backbone can offer a multielectron reaction. On the other hand, the collaborative effect of C=O and C=N bond plays a unique role in the stabilization of anion, while still undeveloped in the design of electrode materials. Actually, a rational devise of different active center in the aromatic system can exert the unexpected effect which cannot be solely achieved by the single active functional group.

To examine the effect of different functional groups on its electrochemical performances, three kinds of pentacene derivatives were investigated as cathode material through the systematic insertion of C=N and C=O bonds into the backbone of pentacene (Figure 1(a)). The correctness of structure has been verified by various characterizations, and consistent with previous report thus could be the evidence for the reliable molecules [23]. Relevant characterizations can be found in [Sec supplementary-material-1]-[Sec supplementary-material-1] and [Sec supplementary-material-1], which include the FTIR, ^1^H-NMR, ^13^C-NMR, MS, elemental analysis, UV-Vis, and XPS. In front of electrochemical test, a prospective study of the structure characteristics of derivatives was performed through the density functional theory (DFT) calculations. For organic semiconductor, the lowest unoccupied molecular orbital (LUMO) energy level is closely related to its redox potential [31, 32]. Compared with other three compounds, TAPQ owns two C=O bonds and four C=N bonds. The synergic integration of these electron-withdrawing groups is expected to induce TAPQ with lower LUMO levels (-3.41 eV) and exhibits higher discharge potentials, which cannot be solely achieved by C=N or C=O bond. Figure 1(b) displays the highest occupied molecular orbital (HOMO) plots of TAPQ and TAPQ^•-^; it can be found that the electron in neutral TAPQ mainly localized around the conjugated system which composed of C=N and C=O bond. However, the delocalization of electron in monoanion TAPQ^•-^ is more uniform and wider on the surface of molecular plane. With the help of conjugated system and the synergic integration of different groups, the reduced species (TAPQ^•-^) realize stabilization and can thus present a theoretical interpretation for the structural superiority of TAPQ. This effect is more obvious in the HOMO plots of TAPQ-4Li, wherein the HOMO plots still retain within the molecular backbone, indicating that the conjugation structure is able to support so much negative charge (Figure 1(b)). When the neutral TAPQ is reduced to monoanion TAPQ^•-^, the natural bond orbital (NBO) charge of nitrogen and oxygen atoms drastically increased and thus will be an active center to bind with the electron-deficient Li cation ([Sec supplementary-material-1], Supporting Information) [33]. Due to the well-designed molecular structure, oxygen and nitrogen atoms in the TAPQ are suitably located for coordination to Li ions, which can efficiently stabilize the negative charge. Such unique chelating coordination environment is impossible in other single active center pentacene derivatives.

To verify the theoretical predictions, the electrochemical behavior of three pentacene derivatives was investigated. Obviously, pristine pentacene cannot deliver any capacity for the lack of any active functional group (Figure 2(a)). In sharp contrast, when carbonyl (C=O) is introduced into pentacene, the resulting 6,13-pentacenequinone (PTQ) exhibits much improved battery performance. The charge/discharge profiles of PTQ contain evident voltage plateaus with average potentials of 2.32 and 1.75 V, respectively (Figure 2(b)). However, the initial discharge capacity of PTQ is only 44 mAh g^−1^ and decays rapidly in the subsequent cycling process and decrease to 33 mAh g^−1^ after 10 cycles.

As a nitrogen-rich heteroacenes, 5,14-dihydro-5,7,12,14-tetraazapentacene (DHTAP) owns a pair of C=N bond in the pentacene skeleton which determines its potential electrochemical performance. The discharge curve of DHTAP is sloping in the potential range of 3.0–2.3 V, but the charge process is relatively complicated, indicating the multistep transformation during the delithiation process (Figure 2(c)). Even though four nitrogen atoms are inserted, the molecular weight of DHTAP (286.12 g mol^−1^) is still lower than that of PTQ (308.08 g mol^−1^). Except a higher theoretical specific capacity, the practical capacity of DHTAP is also superior to PTQ, which embody the advantages of heteroaromatic structure. Although the initial discharge capacity reaches 133 mAh g^−1^, it reduces rapidly at the second cycle and decreases continuously in the succeeding cycles.

It is apparent that the pentacene with C=N bond delivers a higher average discharge/charge voltage and more capacity than the pentacene with C=O bond does. But there is still room for further improvement of electrochemical performances. To a certain extent, DHTAP can be viewed as a phenazine ring connected to a benzene ring by two NH; however, these two sp^3^ hybridization nitrogen atoms break the delocalization of the *π*-system in the pentacene skeleton ([Sec supplementary-material-1], Supporting Information). For this purpose, a compacted multielectron reaction conjugated structure (TAPQ) is developed. On the one hand, TAPQ owns more active sites will provide more capacity. On the other hand, a uniform delocalization of *π* electron on the surface of conjugated molecular skeleton is beneficial to the stabilization of the reduced TAPQ species (Figure 1(b)). Based on the above assumption, the electrochemical behavior of TAPQ was investigated. As the discharge/charge profiles shown in Figure 2(d), the electrochemical behavior of TAPQ is entirely different from that of DHTAP and PTQ. The evident discharge plateau at 2.8 V, 2.1 V and 1.6 V (Figure 2(d)) is well consistent with the reduction peaks in cyclic voltammogram (CV) curves (Figure 2(g)). The overall redox behaviors of differential capacity (dQ/dV) and CV curves are similar and composed of two distinct regions (Figures 2(e) and 2(g)).

To probe the reaction process, the mechanism is investigated from the aspects of reaction kinetics and thermodynamics. As a matter of fact, lithium ion is bound to both the carbonyl oxygen and imine nitrogen during the reduction process. The calculations indicate that a five-membered heterocyclic (C-N-Li-O-C) will be formed during the reduction of TAPQ. NBO charge of TAPQ-Li revealed that even the negative charge on the nitrogen (-0.767) is inferior to oxygen (-0.889) and the interaction is basically equivalent when they bound with lithium ion which possesses a great positive charge (0.882) (Figure 1(c)). These conclusions agree with the analysis of bond length. Except the similar distance from lithium (Li33-O17 1.745 Å; Li33-N3 1.886 Å), the increased bond length of C=O and C=N (Figure 1(c) and [Sec supplementary-material-1], Supporting Information) implies the simultaneous reduction of active bond. Electron density difference map (EDDM) is a useful technique for the characterization of chemical bond. For the synergetic coordination, the evident electronic transfer from oxygen and nitrogen to the neighbor lithium ions is compelling evidence. In Figure 1(c), the electronic density exhibits an accumulation close to lithium ions and depletion around imine nitrogen and carbonyl oxygen, which indicates the obvious interaction between them.

From the viewpoint of stabilization, the monoanionTAPQ^•-^ can be significantly stabilized when the lithium ion is placed in the chelating coordination environment. As shown in Figure 1(c), the coordination energies (Δ*E*_coord,1_ -73.48 kcal mol^−1^) are more remarkable than reduction energies (Δ*E*_red,1_ -48.82 kcal mol^−1^), implying the importance of collaborative coordination effect. Through the synergic integration of different active sites, the simultaneous coordination of N and O to Li (N-Li-O) is more effective than the similar chelating effect of O-Li-O and N-Li-N. Due to the structural symmetry, three possible configurations can be obtained when the second lithium ion inserted (Figure 3(a), [Sec supplementary-material-1]-[Sec supplementary-material-1], Supporting Information). Placing the second lithium ion near the fresh carbonyl oxygen will result in an increased energy. Due to the advantage in energy, two lithium ions binding with the same oxygen (N19-O1-N2) are the most stable configuration.

Thanks to the collaborative coordination center, it is hypothesized that four lithium ions can be stored in TAPQ. This conclusion is supported by thermodynamics calculations wherein the negative stabilization energies value indicates a favorable binding of the fourth lithium ion (Figure 3(b), [Sec supplementary-material-1] and [Sec supplementary-material-1], Supporting Information). Nevertheless, the stepwise binding energies are quite different. Revealed in the CV curves, the first two-reduction wave is overlapped, the third and fourth reduction peak are separated (Figure 2(g)). In fact, careful observation of CV curves found that the first reduction wave consists of two waves and exhibits a strong current density. The reversible four-electron redox process is demonstrated when the CV curves of TAPQ were measured in DMF solutions containing TBAP as electrolytes (Figure 2(f)).

In combination with DFT calculations, ex situ Fourier transform infrared spectroscopy (FTIR) and X-ray photoelectron spectroscopy (XPS) were employed to identify the structural changes. When discharged to 1.3 V, the stretching vibration of C=O bond located at 1696 cm^−1^ disappeared (Figure 4(a)). The vanished C=O bond restored in the next charge process. The stretching vibrations of C=N bonds are observed at 1534, 1512, 1482, 1463 cm^−1^. During the lithiation process, the intensity of C=N bond gradually weakens. In the charge process, these peaks reversibly appear, which suggests the participation of C=N groups. The repeatability FTIR spectral signals in other regions also provide compelling evidence for the reversible electrochemical behaviour of TAPQ. Similarly, the reversible disappearance and formation of the characteristic C=O and C=N bond are also detected in the ex-XPS. In the discharged state, the O 1s band shift toward lower binding energy indicates the participation of C=O in the reaction (Figure 4(b)). Due to the formation of a new bond between N and Li atoms (398.2 eV), the characteristic peak of C=N bond become wider in the discharged state (Figure 4(c)). The simultaneous coordination of oxygen and nitrogen atom to lithium is also confirmed by the C1s spectrum (Figures 4(d)–4(f)). Accompanied by the weakening of C=O and C=N bond, a new peak (290.35 eV) indexed to C–Li appears during discharge process, which results from the lithiation reaction with active groups. In the recharged electrode, C-Li peak disappears and exhibits the reversible transformation of C=O and C=N groups.

More active sites mean more capacity, and TAPQ indeed provides more capacity as expected. The specific discharge and charge capacity of TAPQ are 237 and 232 mAh g^−1^ during the first cycle, respectively (Figure 2(d)). Imperfectly, there is a serious capacity fading in TAPQ when it acts as organic cathode ([Sec supplementary-material-1]-[Sec supplementary-material-1], Supporting Information). As is known, the capacity fading is mainly caused by two reasons [7, 9]. One is the intrinsic electrochemical instability, and the other is the dissolution of active material into the organic electrolyte [34, 35]. To explore the reason of capacity fading, relevant experiments were carried out. Firstly, the disassembled separator becomes green when TAPQ is reduced to anion species which indicates that the dissolution occurred ([Sec supplementary-material-1], Supporting Information). Even though the capacity decays seriously, the discharge/charge profiles maintain well and voltage plateaus are still evident, which indicate the electrochemical behavior of TAPQ is not changed and the electrochemical reaction is still reversible ([Sec supplementary-material-1], Supporting Information). Additionally, the typical FTIR peaks of TAPQ in the cycled electrode are consistent with the pristine samples which further testify the intrinsic chemical stability of TAPQ ([Sec supplementary-material-1], Supporting Information). Therefore, the capacity fading of TAPQ electrode should be attributed to the dissolution rather than its intrinsic electrochemical instability.

In response, coupling TAPQ with graphene will be the possible solution to tackle the dissolution and improve the conductivity. On the one hand, the charge transport of TAPQ is along the *π*–*π* stacking direction of the aromatic rings. As an n-type organic semiconductor, the electron mobility of TAPQ is in the range of 0.04−0.12 cm^2^ V^−1^s^−1^ [36], its intrinsic conductivity is far less than the requirements of high-performance electrode materials. Therefore, it is necessary to introduce conductive carbon and form composite electrodes to solve the poor electrical conductivity. On the other hand, the interactions between organic molecule and graphene are strong physisorption. The introduction of more active group into the pentacene will not only achieve a high capacity but also enhance the binding energies between organic molecule and graphene due to the incorporation of heteroatoms with strong electronegativity. In the dissolution process, an additional energy barrier needs to be overcome when the organic molecule desorbs from graphene, therefore the strong physisorption will slow down the dissolution rate. However, previous reported ultrasonic method is hard to achieve a uniform TAPQ/graphene composite ([Sec supplementary-material-1], Supporting Information).

To tackle the above problems, in situ composite approach was provided. When the DMF solution of TAPQ is quickly injected into ether, TAPQ molecular will undergo a secondary aggregation process and precipitate from the poor solvent. The *π*-conjugated planar structure makes TAPQ has a favor to assemble into 2D structure and thus achieves the transformation from bulk particle to nanoleaf (Figures 5(a) and 5(b)). With the size decreasing to micro/nanometer scale, TAPQ nanoleaf will provide more active sites and facile electronic/ionic transfer and diffusion. Accordingly, when the mixture solution of GO and TAPQ was injected into ether, the leaf-like TAPQ will be formed immediately on the surface of GO. Meanwhile, the smooth GO layer will become shrinkage in the ether, thus coating the TAPQ nanoleaf. In the composite, TAPQ nanoleaf will absorb tightly on the surface of RGO layer for their similar 2D structure (Figures 5(c)-5(d)). Such plane-to-plane contact mode allows a more facilitated electron transfer between TAPQ and graphene layer which is favorable for the stabilization of the reduced TAPQ species. Tethering the electro-active molecules on the surface of RGO through the secondary precipitation method, not only the morphology of TAPQ is changed from the bulk particle to nanoleaf but also allow a closer contact between TAPQ and RGO, thus enhancing the conductivity and lithium-ion accessibility of the electrode, which can get supports from electrochemical impedance spectroscopy ([Sec supplementary-material-1], Supporting Information).

To highlight the superiority of in situ composite method, a series of comparison experiments were carried out. From the start of cycling, the discharge capacity of bulk TAPQ decays rapidly from 238 to 90 mAh g^−1^ at 20th cycle (Figure 6(a)). This situation can be also found in the rate performance, wherein the bulk TAPQ completely lost the capacities when the current density merely increased to 400 mA g^−1^ (Figure 6(b)). Compared with the bulk particle, at each current density, TAPQ nanoleaf all exhibits an enhanced capacity for its large specific surface areas and more exposed active sites. However, there is also a serious capacity fading as that in bulk TAPQ (Figure 6(a)). Fortunately, this problem is solved after the TAPQ is composited with RGO. TAPQ/RGO-S, which obtained from the sonication treatment of RGO and TAPQ, delivers stable capacities of 245, 232, 219, 205, 184, 168, and 161 mAh g^−1^, respectively, as the current densities increased from 50 to 3200 mA g^−1^ (Figure 6(b)). Regretfully, the gradually decreased trend of the capacity can be also found in TAPQ/RGO-S, from the initial discharge at 242 mAh g^−1^ decline to 146 mAh g^−1^ after 100 cycles (Figure 6(a)). In comparison, TAPQ/RGO that prepared from in situ composite route exhibits better electrochemical performance than its sonication treated counterpart. At the same charge/discharge current, the reversible capacity of TAPQ/RGO is higher than that of TAPQ/RGO-S. The discharge capacity of TAPQ/RGO even retained at 221, 205, and 174 mAh g^−1^ when cycled at 800, 1600, and 3200 mA g^−1^ (Figure 6(b)). More importantly, the rapid capacity fading cannot be found in TAPQ/RGO. When cycled at 50 mA g^−1^, the reversible capacity reaches 241 mAh g^−1^ and sustains at 212 mAh g^−1^ at the 100th cycle (Figure 6(a)). As the current density increased to 500 mA g^−1^, the advantage of in situ composite method can be evidently revealed (Figure 6(c)). The reversible capacity of TAPQ/RGO remains at 193 mAh g^−1^ after 2400 cycles with a capacity retention of 80%, whereas TAPQ/RGO-S only achieves at 60% after 1770 cycles. Such a stable cycling performance is rarely seen in organic electrode materials ([Sec supplementary-material-1], Supporting Information).

Encouraged by the superior electrochemical performances presented above and to reveal the potential practical value of TAPQ/RGO, a Li-ion full cell with TAPQ/RGO as the cathode and graphite as the anode was assembled. The discharge/charge behavior of full cell remains the same as that of half-cell, and delivers a reversible capacity of 243 mAh g^−1^ at 50 mA g^−1^ with an average operation voltage of 2.5 V ([Sec supplementary-material-1]a, Supporting Information). The prominent feature of this full cell is the stable cycling performance. Cycled at a constant current of 100 mA g^−1^, the full cell delivers a stable capacity around at 225 mAh g^−1^ and the battery remained 92% of its initial capacity after 220 cycles ([Sec supplementary-material-1]c, Supporting Information).

In summary, to achieve the high energy density and utilization of functional groups, a series of pentacene derivatives are devised through a synergic integration of C=N and C=O bonds into the conjugated skeleton. Based on substantial DFT calculations and characterization techniques, the redox mechanism of TAPQ is investigated from different perspectives. To overcome the high solubility and low conductivity, TAPQ/RGO composite is synthesized through a convenient in situ mixture method and exhibits an ultra-stable cycling stability and a good rate capability. In this work, the concept of functional integration design strategy presents a paradigm on the design of new materials, which not only can achieve the quick discovery of potential high performance organic electrode material but also provide a new strategy on the improvement of battery performance based on the taking full advantage of molecular design. We anticipate that these preliminary investigations will encourage researchers to pay more attention on the structure-performance relationship, thus to have a deeper understanding on the design of organic electrodes.

## Figures and Tables

**Figure 1:**
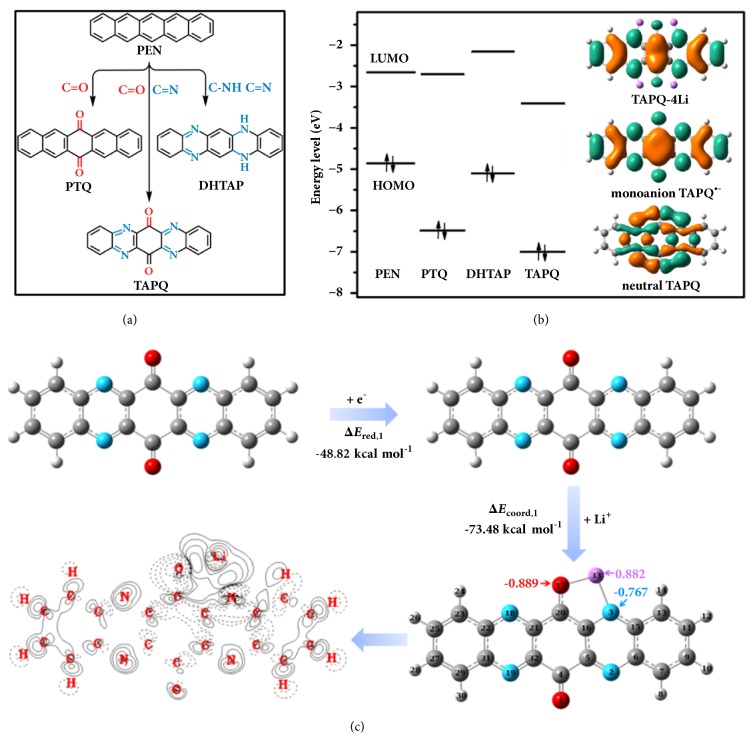
Density functional theory (DFT) calculations and redox mechanism. (a) Illustrated structures of pentacene derivatives studied in this work. (b) Energy level diagram of pentacene derivatives and the HOMO plots of TAPQ, TAPQ^•-^, and TAPQ-4Li. (c) Redox process of TAPQ-Li.

**Figure 2:**
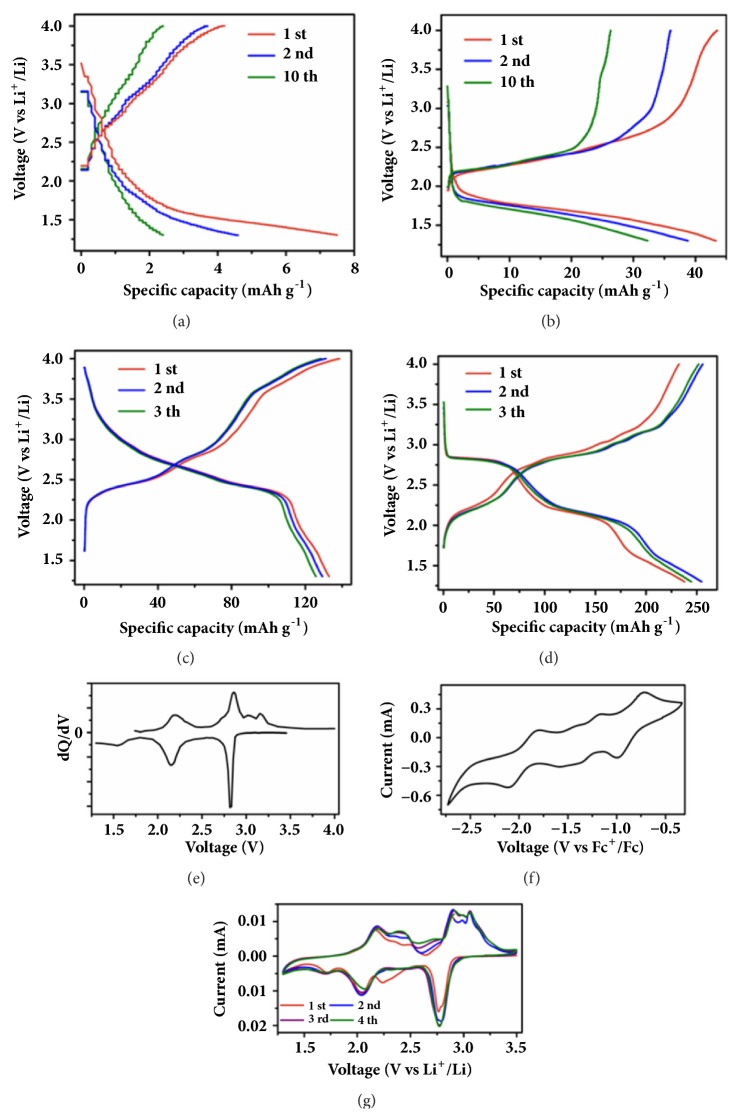
Electrochemical performance of pentacene derivatives. Discharge/charge profiles of (a) PEN, (b) PTQ, (c) DHTAP, and (d) TAPQ in the voltage range of 1.3–4.0 V at a current density of 50 mA g^−1^. (e) Differential capacity (dQ/dV) plots of TAPQ. (f) CV curves of TAPQ measured in DMF solutions containing TBAP as electrolytes. (g) CV curves of TAPQ in coin battery.

**Figure 3:**
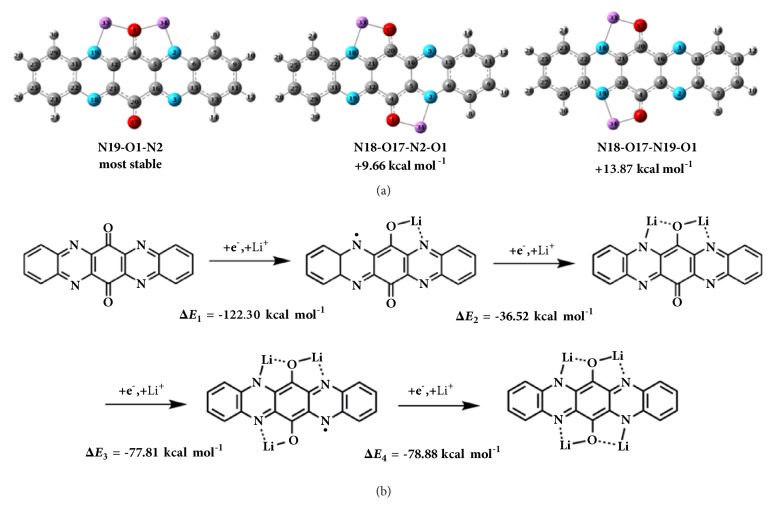
DFT calculation on TAPQ during redox reaction. (a) Three possible configurations of TAPQ-2Li. (b) The stabilization energies (Δ*E*) at various stages of the lithiated TAPQ.

**Figure 4:**
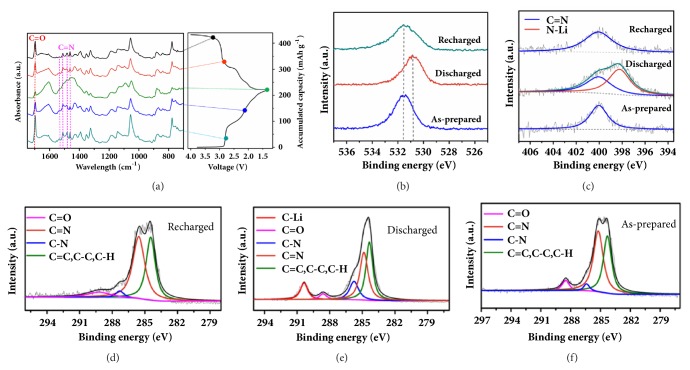
Ex-situ analyses of TAPQ electrode at different states. (a) Ex-situ FTIR characterizations. (b) Ex-situ XPS local scan spectra of O 1s regions. (c) Ex-situ XPS local scan spectra of N 1s regions. (d-f) Ex-situ XPS local scan spectra of C 1s regions.

**Figure 5:**
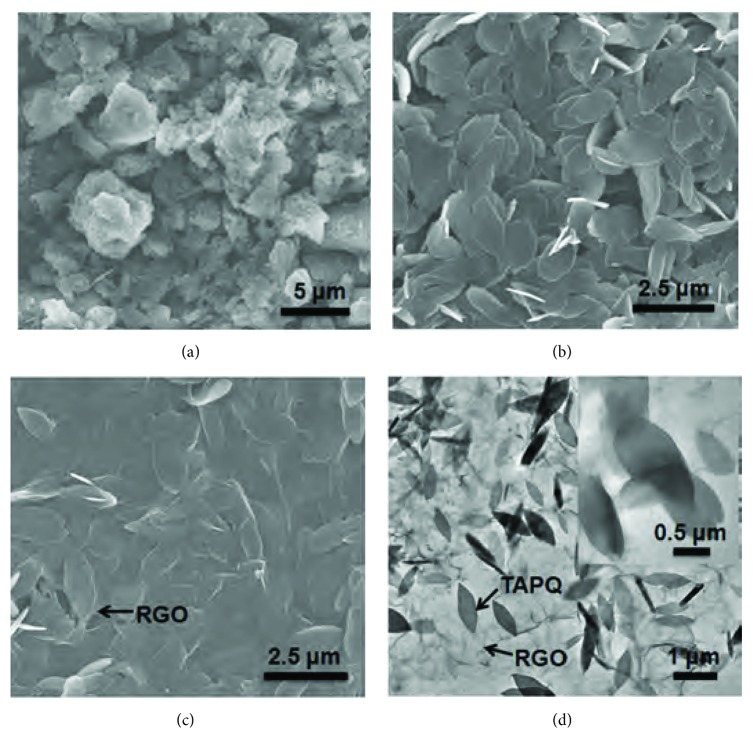
Morphology of TAPQ and its composite. (a) SEM images of bulk TAPQ. (b) SEM images of TAPQ nanoleaf. (c) SEM images of TAPQ/RGO. (d) TEM images of TAPQ/RGO.

**Figure 6:**
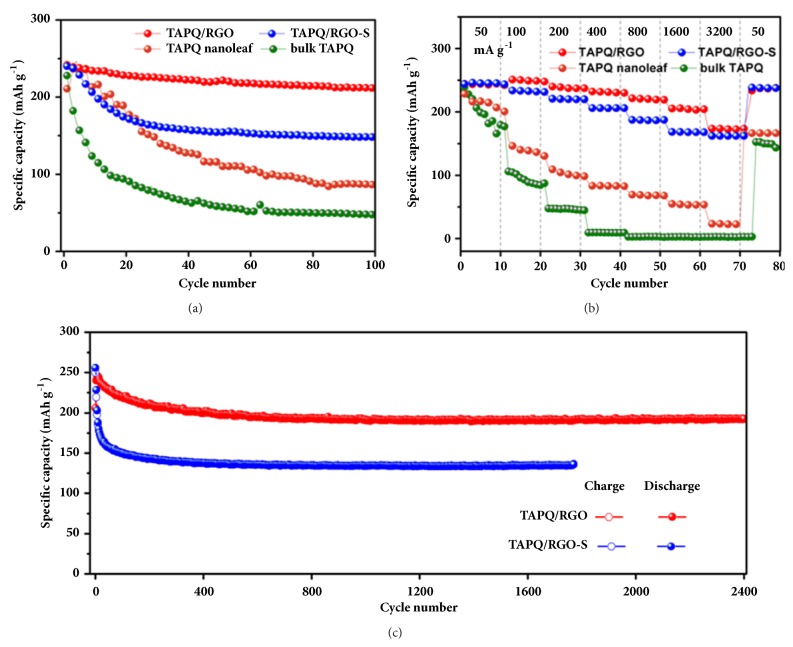
Electrochemical performance of TAPQ and its composites. (a) Cycling performance of TAPQ and its composites at a current density of 50 mA g^−1^. (b) Rate performance of TAPQ and its composites. (c) Long-term cycling stability of TAPQ/RGO and TAPQ/RGO-S at a current density of 500 mA g^−1^.

## Supplementary Materials

Supplementary Materials Scheme S1. Synthesis routes of TAPQ. Scheme S2. Synthesis routes of PTQ.** Figure S1**. (a) FTIR spectral of PTQ. (b) XRD pattern of PTQ.** Figure S2**. ^1^H NMR (DMSO-d_6_) spectrum of DHTAP.** Figure S3**. FTIR spectra of DHTAP.** Figure S4.** ESI-FTMS of DHTAP.** Figure S5**. ^1^H NMR (DMSO-d_6_) spectrum of TAPQ.** Figure S6**. ^13^C NMR (DMSO-d_6_) spectrum of TAPQ.** Figure S7**. ESI-FTMS of TAPQ.** Table S1.** Elemental analysis results of pentacene derivatives and TAPQ/RGO.** Figure S8.** UV-vis spectra of TAPQ.** Figure S9**. XPS spectra of the C1s, O1s and N1s peak fittings of TAPQ.** Figure S10.** FTIR of bulk TAPQ, TAPQ nanoleaf and TAPQ/RGO.** Figure S11.** TGA of TAPQ and composite.** Figure S12. **The natural bond orbital (NBO) charge distributions of (a) the neutral TAPQ and (b) monoanion TAPQ^•-^.** Figure S13. **(a) The highest occupied molecular orbital (HOMO) plot of DHTAP. (b) The lowest unoccupied molecular orbital (LUMO) plot of DHTAP.** Figure S14.** The possible configurations of TAPQ-2Li: (a, d) the optimized structures; (b, e) HOMO plots; (c, f) LUMO plots of N18-O17-N19-O1 and N18-O17-N2-O1.** Figure S15.** The most stable configurations of TAPQ-2Li: (a) the optimized structures; (b) HOMO plots; (c) LUMO plots of N19-O1-N2.** Figure S16.** (a, d) the optimized structures; (b, e) HOMO plots; (c, f) LUMO plots of TAPQ-3Li and TAPQ-4Li.** Table S2.** The major bond length change in TAPQ intermediate. The unit of bond length is Å.** Table S3. **Summary of energies of the redox intermediate and reaction step.** Figure S17.** (a) The FTIR spectrum of cycled TAPQ electrode and pristine bulk TAPQ. The insets are the optical photograph of cycled separator. The left one corresponds to the bulk TAPQ electrode. The right one corresponds to the TAPQ/RGO electrode. (b) The solubility of TAPQ (left), TAPQ/RGO (right) in the electrolyte.** Figure S18.** Discharge/charge profiles of TAPQ nanoleaf at current density of 100 mA g^−1^.** Figure S19. **SEM image of TAPQ/RGO-S.** Figure S20.** Electrochemical impedance spectroscopy of TAPQ and its composites: (a) TAPQ nanoleaf and bulk TAPQ; (b) TAPQ/RGO and TAPQ/RGO-S after the electrode discharged to 1.3 V at 5^th^ cycle; (c) TAPQ/RGO at 50^th^ and 100^th^ cycle; (d) TAPQ/RGO-S at 50^th^ and 100^th^ cycle.** Figure S21: **Electrochemical performances of TAPQ/RGO half-cell. (a) CV curves of the initial 3 cycles at 0.1 mV s^−1^; (b) CV curves of TAPQ/RGO at different scan rates; (c) log(i) versus log(v) plots at different redox states.** Figure S22.** Charge/discharge curves of TAPQ/RGO (a) and RGO (b) at a current density of 50 mA g^−1^ in the voltage range of 1.3-4.0 V.** Figure S23. **(a) Charge/discharge curves of TAPQ/RGO full cell in the voltage range of 1.29 and 3.65 V at 50 mA g^−1^; (b) Charge/discharge curves of graphite cycling between 0.01 and 1.4 V at 50 mA g^−1^; (c) Cycling performance of LIBs full cell by using a TAPQ/RGO cathode and graphite anode at 100 mA g^−1^.** Figure S24. **SEM images of TAPQ/RGO electrodes: a) after 50 cycles, b) after 100 cycles. Comparing the electrode after cycling, the nanoleaf morphology of TAPQ is still maintained and well composited with RGO and thus demonstrating the superiority of in situ composite method.** Figure S25. **Voltage-Capacity curves of TAPQ and its composites corresponding to Figures 6(a) and 6(b) at the current density of 50 mA g^−1^.** Table S4. **Comparison of TAPQ/RGO with those organic/carbon composite electrode materials.
