# Supplementary material for: Achieving of High Density/Utilization of Active Groups via Synergic Integration of C=N and C=O Bonds for Ultra-Stable and High-Rate Lithium-Ion Batteries
Source: Research (Wash D C). 2018 Dec 16;2018:1936735. doi: 10.1155/2018/1936735 (PMC6750095; doi:10.1155/2018/1936735)
Supplement: Supplementary Materials — Scheme S1. Synthesis routes of TAPQ. Scheme S2. Synthesis routes of PTQ. Figure S1. (a) FTIR spectral of PTQ. (b) XRD pattern of PTQ. Figure S2. 1H NMR (DMSO-d6) spectrum of DHTAP. Figure S3. FTIR spectra of DHTAP. Figure S4. ESI-FTMS of DHTAP. Figure S5. 1H NMR (DMSO-d6) spectrum of TAPQ. Figure S6. 13C NMR (DMSO-d6) spectrum of TAPQ. Figure S7. ESI-FTMS of TAPQ. Table S1. Elemental analysis results of pentacene derivatives and TAPQ/RGO. Figure S8. UV-vis spectra of TAPQ. Figure S9. XPS spectra of the C1s, O1s and N1s peak fittings of TAPQ. Figure S10. FTIR of bulk TAPQ, TAPQ nanoleaf and TAPQ/RGO. Figure S11. TGA of TAPQ and composite. Figure S12. The natural bond orbital (NBO) charge distributions of (a) the neutral TAPQ and (b) monoanion TAPQ•-. Figure S13. (a) The highest occupied molecular orbital (HOMO) plot of DHTAP. (b) The lowest unoccupied molecular orbital (LUMO) plot of DHTAP. Figure S14. The possible configurations of TAPQ-2Li: (a, d) the optimized structures; (b, e) HOMO plots; (c, f) LUMO plots of N18-O17-N19-O1 and N18-O17-N2-O1. Figure S15. The most stable configurations of TAPQ-2Li: (a) the optimized structures; (b) HOMO plots; (c) LUMO plots of N19-O1-N2. Figure S16. (a, d) the optimized structures; (b, e) HOMO plots; (c, f) LUMO plots of TAPQ-3Li and TAPQ-4Li. Table S2. The major bond length change in TAPQ intermediate. The unit of bond length is Å. Table S3. Summary of energies of the redox intermediate and reaction step. Figure S17. (a) The FTIR spectrum of cycled TAPQ electrode and pristine bulk TAPQ. The insets are the optical photograph of cycled separator. The left one corresponds to the bulk TAPQ electrode. The right one corresponds to the TAPQ/RGO electrode. (b) The solubility of TAPQ (left), TAPQ/RGO (right) in the electrolyte. Figure S18. Discharge/charge profiles of TAPQ nanoleaf at current density of 100 mA g−1. Figure S19. SEM image of TAPQ/RGO-S. Figure S20. Electrochemical impedance spectroscopy of TAPQ and its composites: (a) TAP [file 1936735.f1.pdf]

Supplementary Materials

Achieving of High Density/Utilization of Active Groups via Synergic Integration of C=N and C=O Bonds for Ultra-Stable and High-Rate Lithium-Ion Batteries

Experimental Section

1. Materials

2,5-Dihydroxy-1,4-benzoquinone (AR, TCI, Japan), 1,4-Cyclohexanedione (98%, AR, Aladdin), *o*-Phthalaldehyde (98%, AR, Aladdin), 1,2-Phenylediamine(AR, Aladdin), Potassium dichromate ($K_2Cr_2O_7$, AR, Beijing Chemical Works, China), Graphite powder (40 nm, 99%, Aladdin), Sulfuric acid (H_2SO_4, 98%, Beijing Chemical Works, China), KOH (AR, Beijing Chemical Works, China), Pentacene (PEN, 97%, AR, Aladdin), Graphite (Beijing Chemical Works, China), Nitric acid (HNO_3, Beijing Chemical Works, China), $KMnO_4$ (AR, Beijing Chemical Works, China), Hydrogen peroxide (H_2O_2, XilongChemicals), PVDF (Arkema HSV900), Acetylene black (Hong-xin Chemical Works), N-Methyl-2-pyrrolidone (NMP, 99%, Aldrich), Acetone(AR, Beijing Chemical Works, China), N,N-Dimethylformamide(DMF, SinopharmChemical Reagent, AR), Ether (SinopharmChemical Reagent, AR), Glucose (AR, Aladdin), Ammonia solution (XilongChemicals, 25% w/w), Commercial electrolyte LB-301 (ZhangjiagangGuotaiHuarong Chemical New Material, 1 M $LiPF_6$ in a 1:1 mass ratio mixture of ethylene carbonate and dimethyl carbonate), Glass fiber (GF, Whatman), Ethanol (99.7%, AR, Beijing Chemical Works, China), All the reagents used in the experiment were of analytical grade purity and were used as received. De-ionized water with the specific resistance of 18.2 MΩ·cm was obtained by reversed osmosis followed by ion-exchange and filtration.

2. Sample synthesis

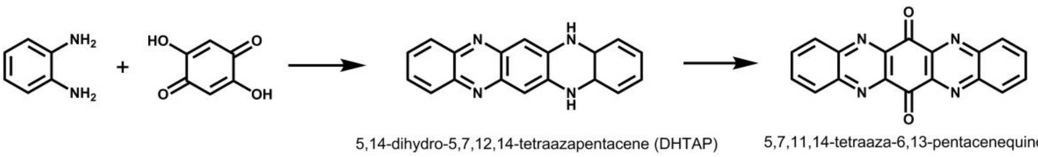

5,14-dihydro-5,7,12,14-tetraazapentacene (DHTAP)   5,7,11,14-tetraaza-6,13-pentacenequinone (TAPQ)

Scheme S1. Synthesis routes of TAPQ

2.1 Synthesis of 5,14-dihydro-5,7,12,14-tetraazapentacene (DHTAP)[S1]

2,5-dihydroxy-1,4-benzoquinone and 1,2-phenylediaminewere mixed in a stoichiometric ratio (1:5 mol ratio) and ground together with a mortar and pestle. The resulted mixture was heated to 160℃ for 5 hour under Ar atmosphere and allowed to cool down room temperature,

naturally. The crude product was repeatedly washed with acetone and filtered to yield the purple powder.

2.2 Synthesis of 5,7,11,14-tetraaza-6,13-pentacenequinone (TAPQ)

808 mg (2.84 mmol) of 5,14-dihydro-5,7,12,14-tetraazapentacene was added to the solution of H_2SO_4 (25 mL concentrated sulfuric acid in 100 mL water). Then, 4g (13.6 mmol) of potassium dichromate was slowly added into the mixture solution and refluxed for 1 hour. Finally, the hot solution was poured into 200 mL of ice-water. The resulting yellow powder was filtered and washed with water and acetone subsequently. This washing step was repeated three times, and the solid was dried under vacuum at 80 ºC for 12 hour.

2.3 Synthesis of TAPQ nanoleaf

The obtained TAPQ sample was put into DMF and sonicated for a few minutes, and the soluble part was further collected through centrifugation. Then, saturated TAPQ solution was quickly injected into the stirred ether solution. After injection, yellow insoluble precipitate was formed immediately. The final product was obtained through centrifugation and dried.

2.4 Synthesis of TAPQ/RGO

GO is synthesized following the literature procedure.[S2] GO was dispersed in DMF solution, and then mixed with saturated TAPQ solution. The mass ratio of GO and TAPQ is 2:1. The mixture solution was injected into the stirred ether solution. The yellow insoluble flocculent material was then centrifugated and washed repeatedly by shaking with ethanol. Finally, TAPQ/GO was reduced by glucose.[S3] The remaining solid material can be obtained through centrifugation and dried under vacuum at 60 ºC.

2.5 Synthesis of 6,13-pentacenequinone (PTQ)[S4]

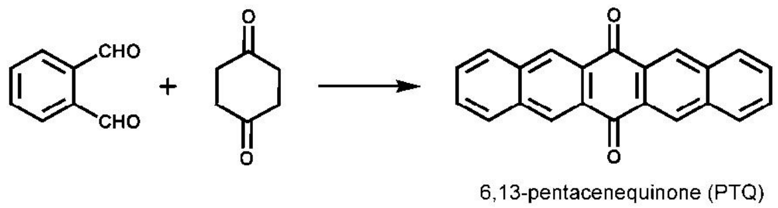

Scheme S2. Synthesis routes of PTQ

1,4-Cyclohexanedione (5.6 g, 0.05 mol) and *o*-phthalaldehyde (15.0 g, 0.11 mol) were added in 80 mL ethanol at room temperature for 1h and produce a yellow solution. Then, KOH solution (2.8 g KOH dissolved in 80 mL water) was slowly added into the mixture of

1,4-cyclohexanedione and *o*-phthalaldehyde, a large amount of precipitation can be observed. After stirred in ice bath for 0.5 hour, the mixture was turn to reflux for 7 hour. The prepared yellow solid was collected by reduced pressure filtration, washed repeatedly by shaking with methanol and water, and dried in vacuum at 100 ºC overnight. After recrystallization from DMF for twice, the purity of PTQ can be improved.

3. Characterization

SEM was performed on a field emission Hitachi S-4800 instrument operated at an accelerating voltage of 10 kV. TEM was performed with a FEI Tecnai G2 F20-Twin instrument having a field-emission gun operated at 200 kV. XPS spectra were obtained with an ESCALAB MK II X-ray photoelectron spectrometer with an Al Kα source. The NMR spectra were obtained on a BrukerAvance III HD500 MHz NMR spectrometer. Fourier transform infrared spectroscopy (FTIR) measurements were performed on a Bruker IFS 66V/S spectrometer using ATR-FTIR technology. The UV−vis spectra were obtained on a TU-1901 spectrophotometer (Persee, China).

4. The preparation procedure of battery and electrochemical measurement

The cathode consisted of 80wt% active compounds, 10wt% acetylene black and 10wt% PVDF. It should be noted that the specific capacity of TAPQ/RGO and TAPQ/RGO-S is calculated based on the mass of TAPQ in the composite. The mixture was dispersed in NMP to form slurry. Then, the slurry was spread onto Al foil with the help of automatic coater, followed by subsequent drying in vacuum (80 °C, 24 h) to remove the traces of solvent molecules. The electrodes were then pressed and cut into disks before transferred into glove box. Coin cells (CR2025) were assembled in the laboratory by using lithium metal as counter electrode, GF membrane as separator. For Li-ion batteries, the electrolyte was commercial LB-301 (1 M $LiPF_6$ in EC-DMC (1:1 w/w)). The active material loading amount is around at 1-2 mg cm^{-2}. The anode was prepared by mixing graphite and PVDF together in 80:20 weight ratio (with Cu foil as current collector). Afteroptimizingthe proportion of cathode/anode, the mass ratio between them is set at 1.4:1. The full cells were assembled with pre-lithiatedgraphiteanode and TAPQ cathode. The graphite anode was coupled with lithium foil as counter electrode, and pre-treated by discharge/charge during 0.01 and 1.4 V at 50 mA g^{-1} for 10 cycles when the Coulombic efficiency was close to 100 % (the open-circuit voltage is around at 2.9 V versus lithium). Then, the graphite anode was discharged to 0.01 V and disassembled in argon-filled glove box. The full cells were assembled by using pre-lithiated

graphite anode and pristine TAPQ cathode, GF membrane as separator. Galvanostatic charge–discharge cycling tests were performed with a Land CT2001A battery testing system (Land, P. R. China) at room temperature. Impedance and the cyclic voltammetry measurements were performed with a VMP3 electrochemical workstation (Bio-Logic, France). Electrochemical impedance spectroscopy was performed within 10^5–10^{-1} Hz.

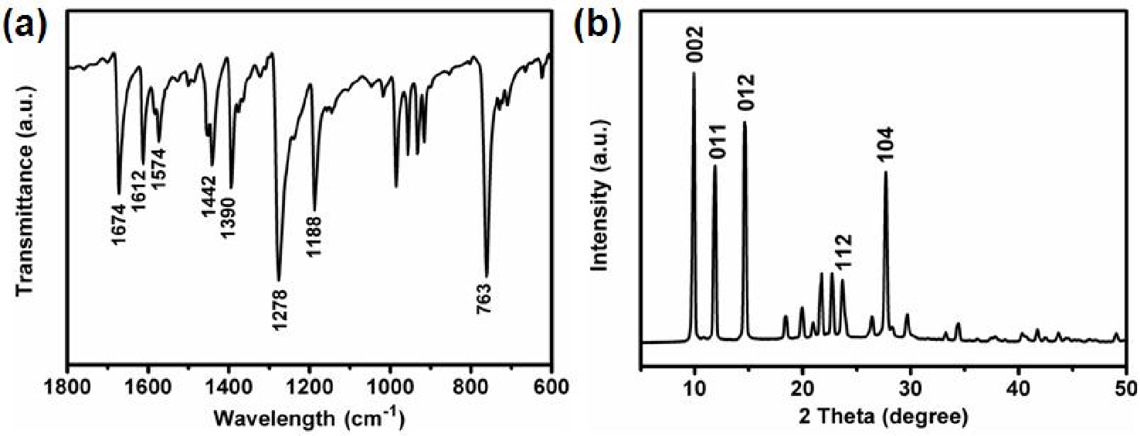

Figure S1. (a) FTIR spectral of PTQ. (b) XRD pattern of PTQ.

The structure of PTQ can be verified by the FTIR spectral, wherein the C=O peak can be found at 1674 cm^{-1}. The peak at 1612 and 1574 cm^{-1} corresponds to the stretching of benzene ring. The diffraction peaks at 9.8, 11.9, 14.7, 23.6, and 27.7° match (002), (011), (012), (112), and (104) reflection planes, respectively (JCPDS card no. 47-2123).

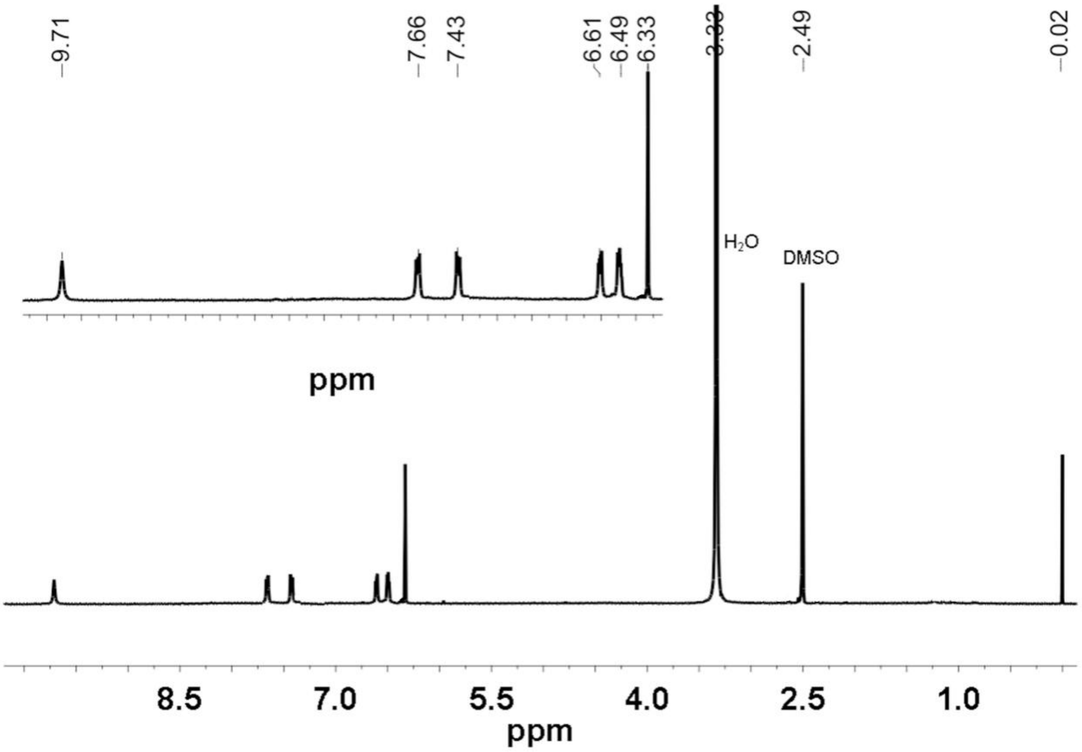

Figure S2. ^{1}H NMR (DMSO-d$_6$) spectrum of DHTAP.

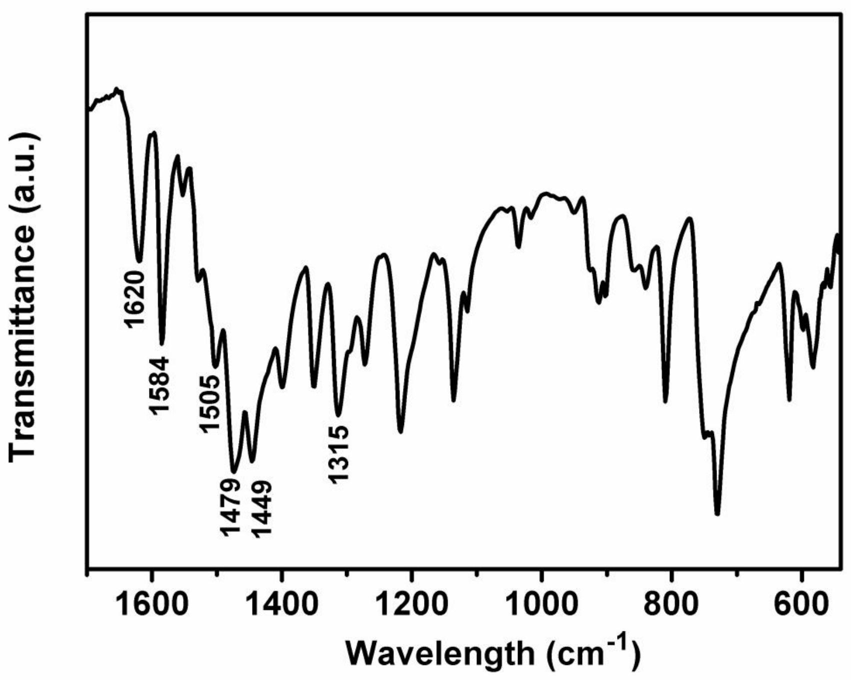

Figure S3. FTIR spectra of DHTAP.

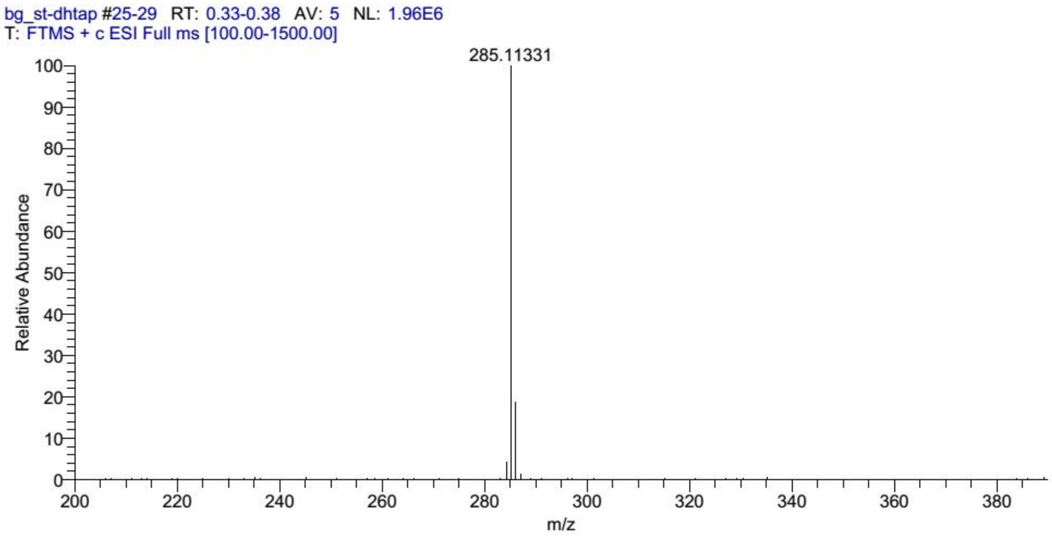

Figure S4. ESI-FTMS of DHTAP.

Calculated for $[DHTAP+H]^{+}$: 285.11347, found: 285.11331.

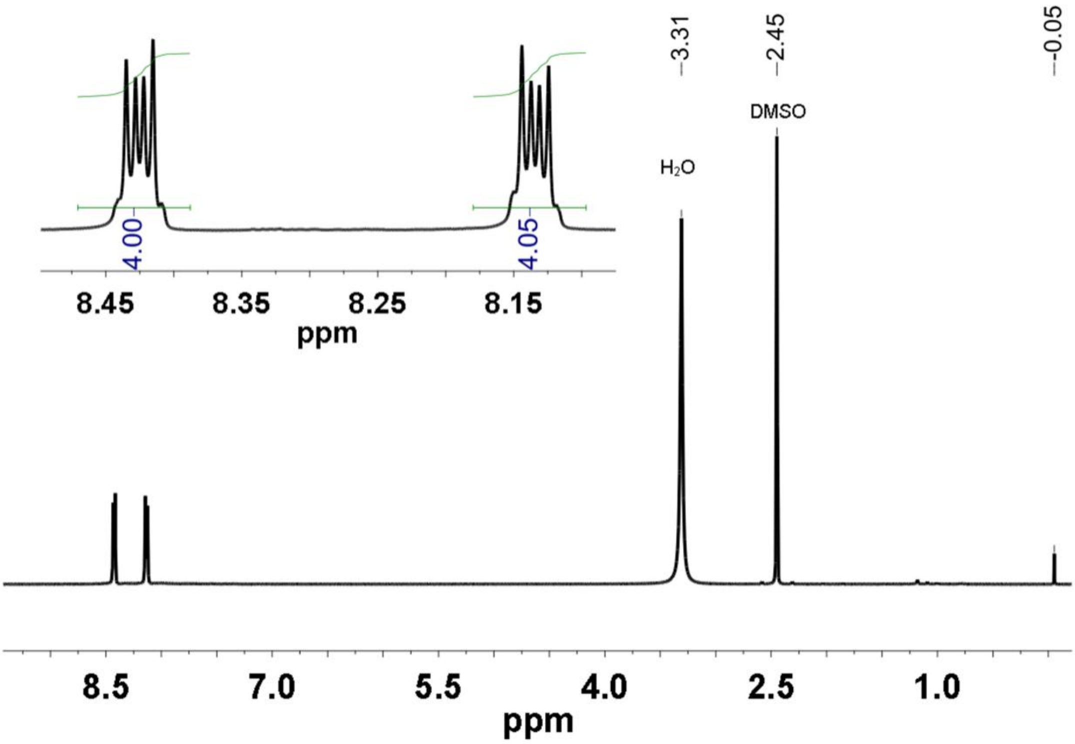

Figure S5. ^{1}H NMR (DMSO-d_6) spectrum of TAPQ.

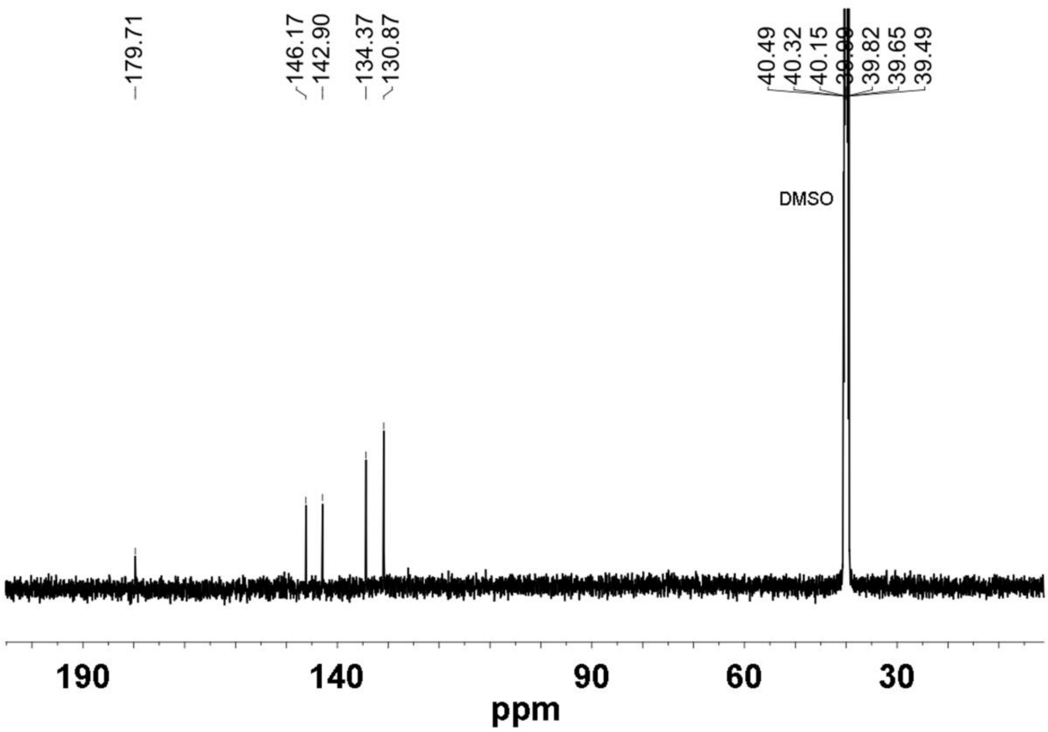

Figure S6. ^{13}C NMR (DMSO-d_6) spectrum of TAPQ.

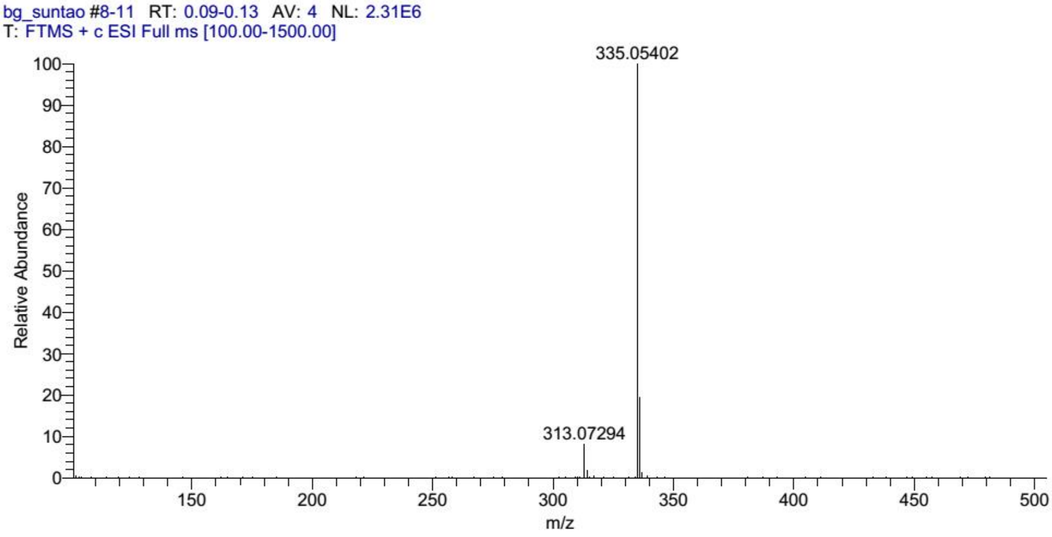

Figure S7. ESI-FTMS of TAPQ.

Calculated for $[TAPQ+Na]^+$: 335.05395, found: 335.05402.

Table S1. Elemental analysis results of pentacene derivatives and TAPQ/RGO.

Sample		N	C	H	O	C/N	C/O	N/O	C/H
TAPQ	Calculated	17.95	69.23	2.56	10.26	3.86	6.75	1.75	27.04
	Found	17.89	69.15	2.73	10.21	3.87	6.77	1.75	25.33
DHTAP	Calculated	19.71	76.06	4.23		3.89			17.98
	Found	19.53	75.85	4.30		3.88			17.64
PTQ	Calculated		85.70	3.90	10.39		8.25		21.97
	Found		85.47	4.19	10.38		8.23		20.40

Elemental analysis clearly illustrates the formation of pentacene derivatives (TAPQ, DHTAP, and PTQ). For example, the measured C, H, O and N contents in TAPQ are respectively, 69.15 %, 2.73 %, 10.21 % and 17.89 %, indicating that the C/N, C/O, and N/O molar ratios are respectively, 3.87, 6.77 and 1.75, which are roughly consistent with the theoretical values of 3.86, 6.75 and 1.75.

**Sample**	**N**	**C**	**H**
RGO	0.01	39.76	2.260
TAPQ/RGO	13.23	58.90	2.637

The content of TAPQ is caculated based on the N contents:

$TAPQ\% = [(composite_{N\ contents} - RGO_{N\ contents})/(TAPQ_{found\ N\ contents})]*100\ \%$.

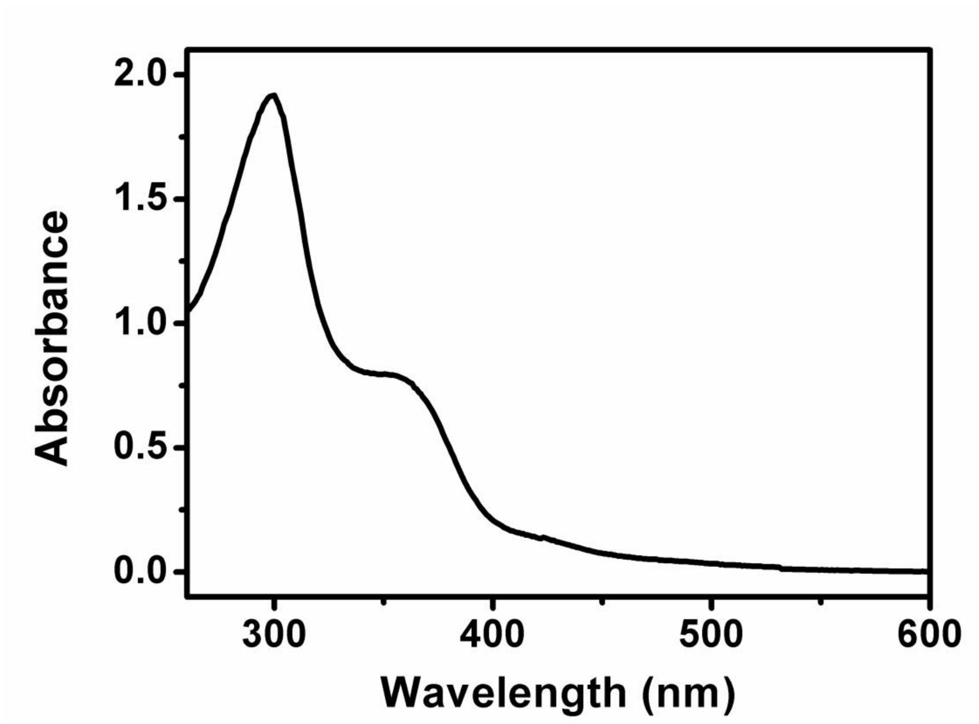

Figure S8. UV-vis spectra of TAPQ.

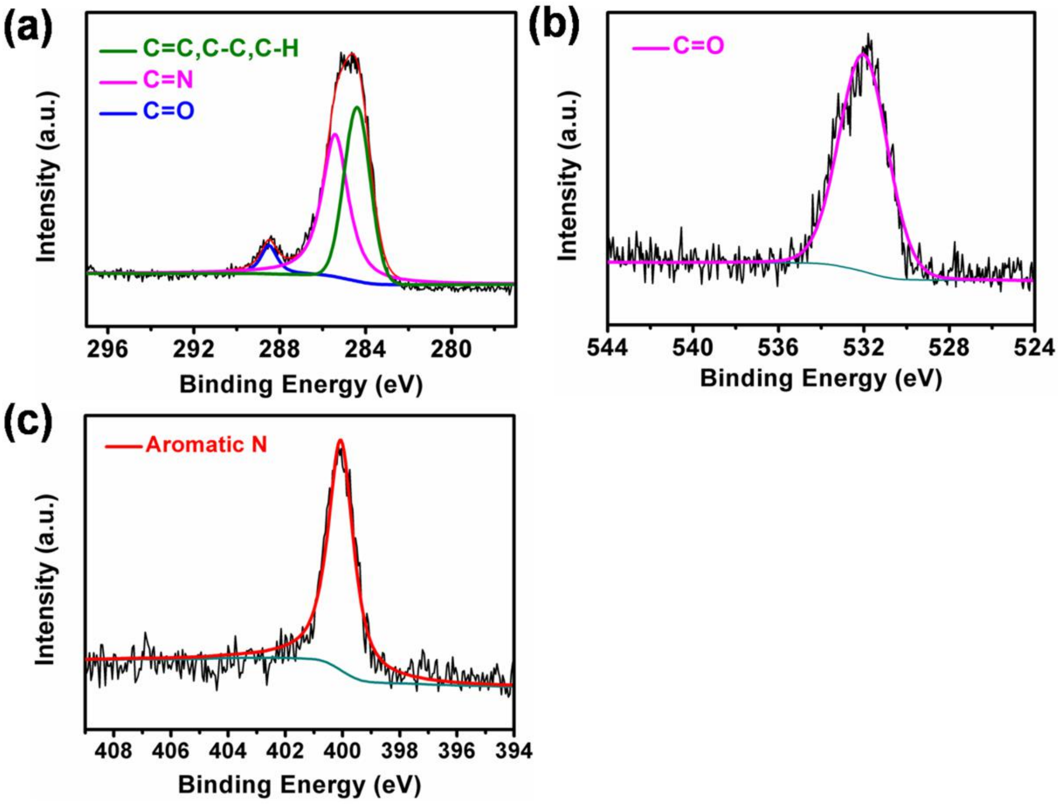

Figure S9. XPS spectra of the C1s, O1s and N1s peak fittings of TAPQ.

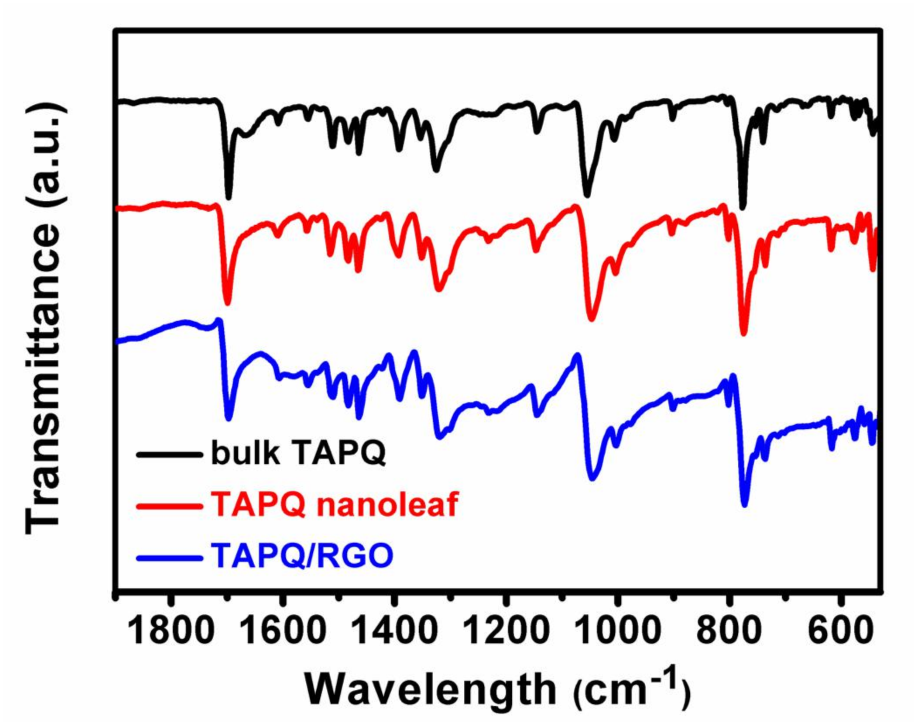

Figure S10. FTIR of bulk TAPQ, TAPQ nanoleaf and TAPQ/RGO.

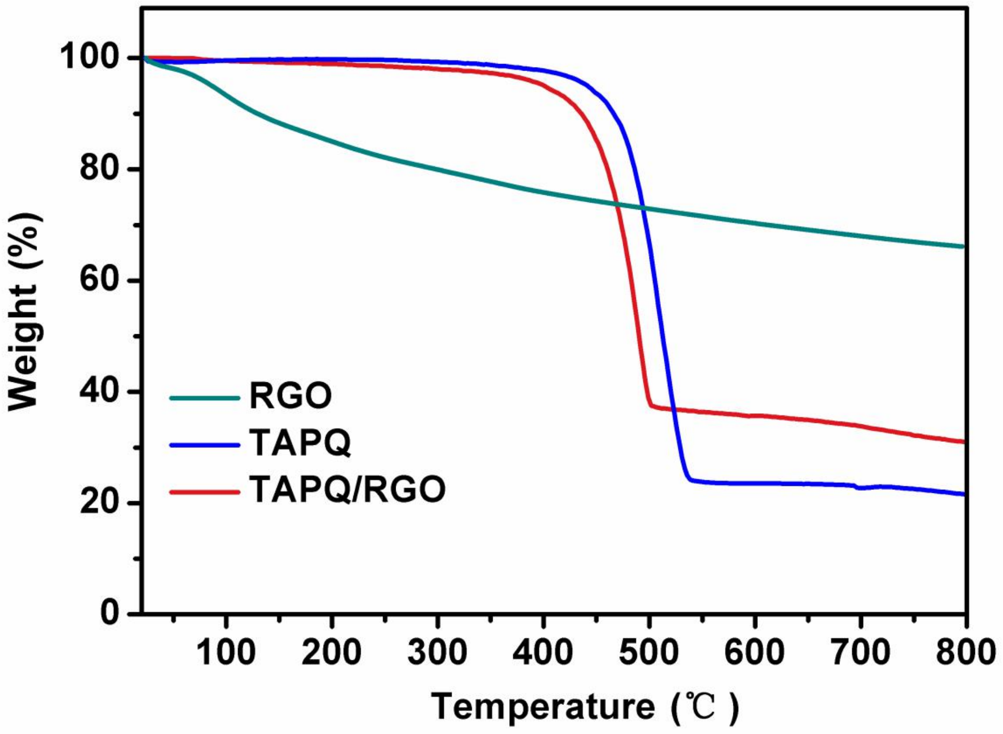

Figure S11. TGA of TAPQ and composite.

The content of TAPQ is caculated as follow:

TAPQ %= $[(RGO_{Weight\ retention\ ratio}-composite_{Weight\ retention\ ratio})/(RGO_{Weight\ retention\ ratio}-TAPQ_{Weight\ retention\ ratio})]*100\ \%$.

The mass percentage of TAPQ in the composite was determined to be 74 % by the elemental analysis (Table S1) and TGA (Figure S11).

Theoretical calculations and related results.

DFT calculations were performed with the Gaussian 09 program. All geometry optimizations were carried out at the B3LYP method with the 6-311G(d,p) basis set.

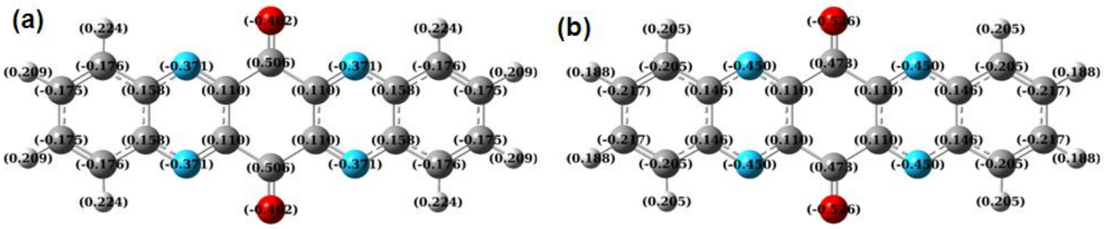

Figure S12. The natural bond orbital (NBO) charge distributions of (a) the neutral TAPQ and (b) monoanion TAPQ$^{\bullet-}$.

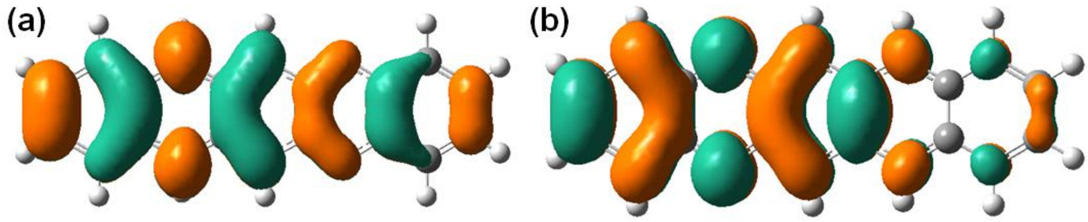

Figure S13. (a) The highest occupied molecular orbital (HOMO) plot of DHTAP. (b) The lowest unoccupied molecular orbital (LUMO) plot of DHTAP.

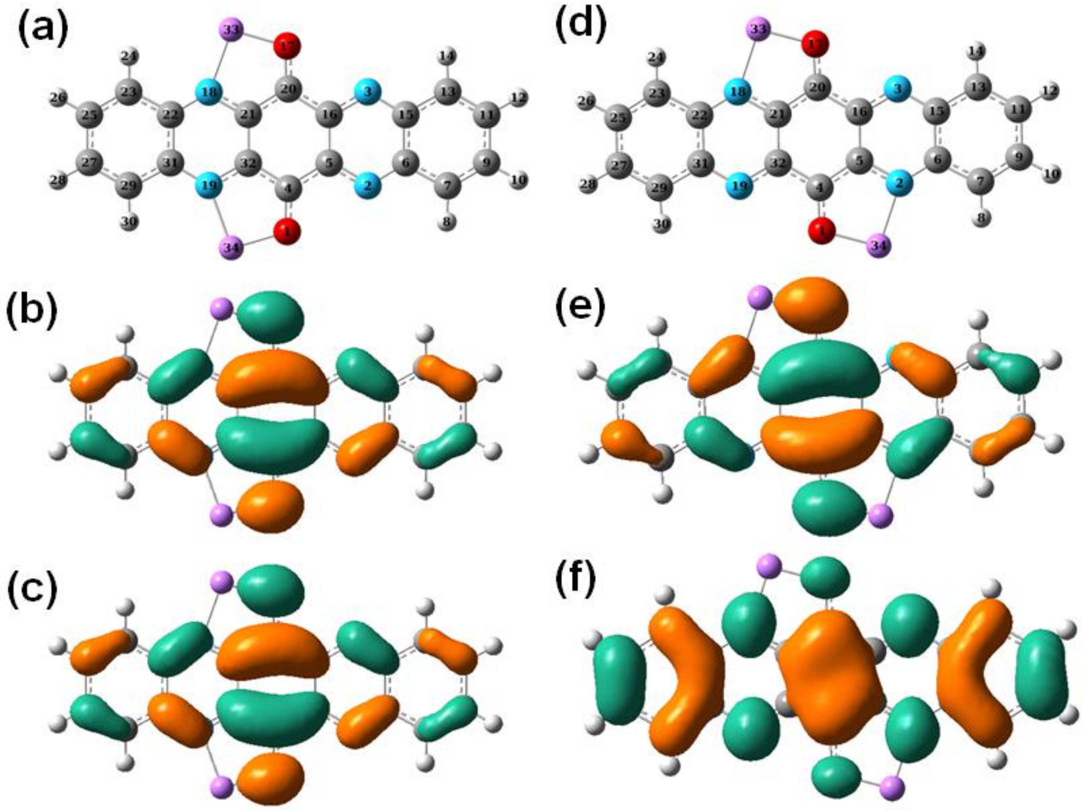

Figure S14. The possible configurations of TAPQ-2Li: (a, d) the optimized structures; (b, e) HOMO plots; (c, f) LUMO plots of N18-O17-N19-O1 and N18-O17-N2-O1.

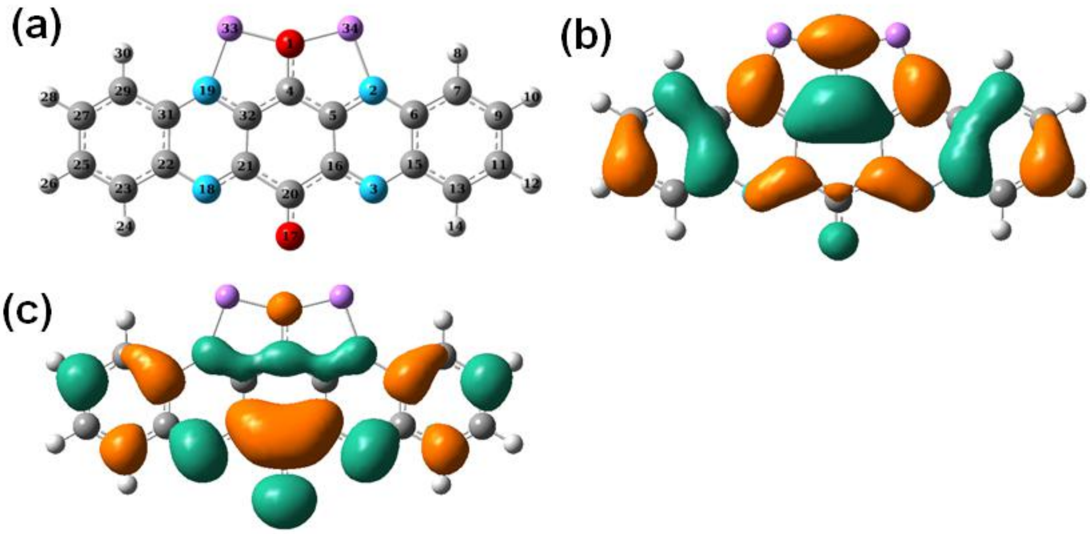

Figure S15. The most stable configurations of TAPQ-2Li: (a) the optimized structures; (b) HOMO plots; (c) LUMO plots of N19-O1-N2.

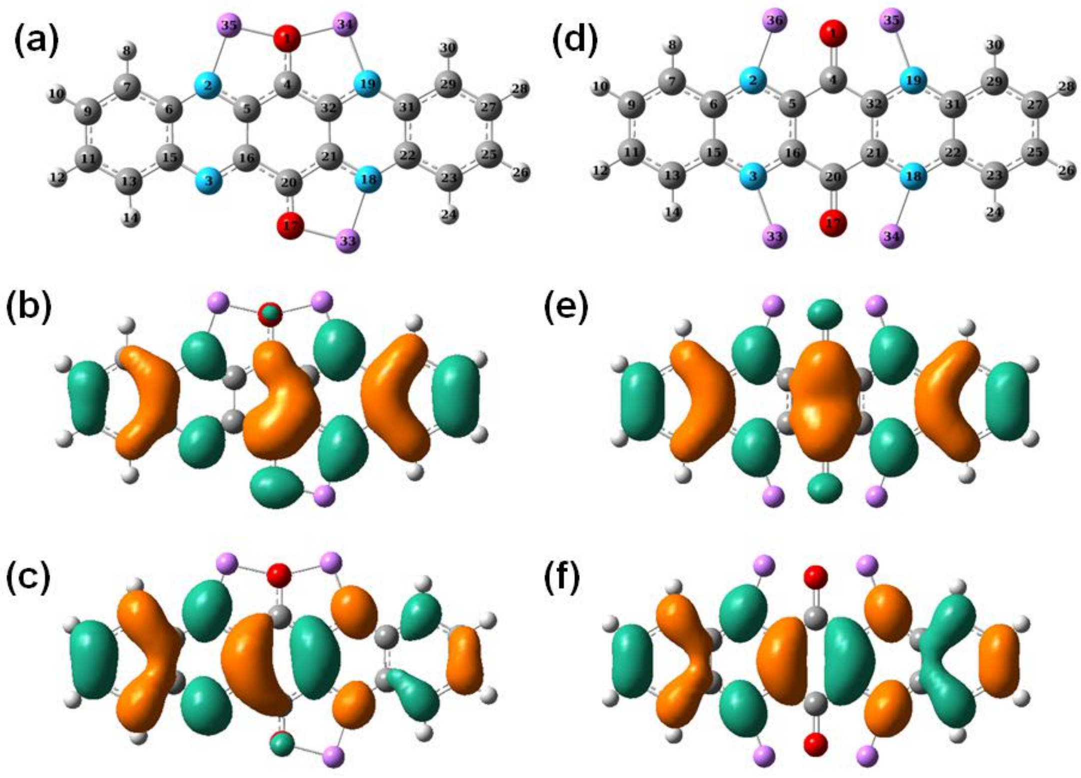

Figure S16. (a, d) the optimized structures; (b, e) HOMO plots; (c, f) LUMO plots of TAPQ-3Li and TAPQ-4Li.

Table S2. The major bond length change in TAPQ intermediate. The unit of bond length is Å.

	C20-O17	C4-O1	N3-C16	C16-C20	Li33-N3	Li33-O17
TAPQ	1.206	1.206	1.319	1.510		
TAPQ-1Li	1.304	1.210	1.390	1.419	1.886	1.745
TAPQ-2Li	1.364	1.209	1.365	1.409	1.899	1.787
TAPQ-4Li	1.373	1.373	1.356	1.406	1.894	1.782

Table S3. Summary of energies of the redox intermediate and reaction step.

Energy	Hartree	eV	kcal/mol
$E_{(\mathrm{Li})}$	−7.4909		
$E_{(\mathrm{TAPQ})}$	-1060.4226		
$E_{(\mathrm{TAPQ{\bullet}-})}$	-1060.5004		
$E_{(\mathrm{TAPQ\text{-}1Li})}$	-1068.1084		
$E_{(\mathrm{TAPQ\text{-}2Li})}$	-1075.6575		
$E_{(\mathrm{TAPQ\text{-}3Li})}$	-1083.2724		
$E_{(\mathrm{TAPQ\text{-}4Li})}$	-1090.8890		
ΔE_1(for 1Li)	-0.1949	-5.3035	-122.3017
ΔE_2(for 2Li)	-0.0582	-1.5837	-36.5211
ΔE_3(for 3Li)	-0.1240	-3.3742	-77.8112
ΔE_4(for 4Li)	-0.1257	-3.4205	-78.8780
$\Delta E_{(\mathrm{red,1})}$	-0.0778	-2.1170	-48.8202
$\Delta E_{(\mathrm{coor,1})}$	-0.1171	-3.1865	-73.4814

The stabilization energies were defined as ΔE:

$\Delta E(\text{for 1Li})=\Delta E_1=E_{(\mathrm{TAPQ\text{-}1Li})}-E_{(\mathrm{TAPQ})}-E_{(\mathrm{Li})}$;

$\Delta E(\text{for 2Li})=\Delta E_2=E_{(\mathrm{TAPQ\text{-}2Li})}-E_{(\mathrm{TAPQ\text{-}1Li})}-E_{(\mathrm{Li})}$;

$\Delta E_{(\mathrm{red,1})}=E_{(\mathrm{TAPQ{\bullet}-})}-E_{(\mathrm{TAPQ})}$;

$\Delta E_{(\mathrm{coor,1})}=E_{(\mathrm{TAPQ\text{-}1Li})}-E_{(\mathrm{TAPQ{\bullet}-})}-E_{(\mathrm{Li})}$ where $E_{(\mathrm{TAPQ\text{-}2Li})}$, $E_{(\mathrm{TAPQ\text{-}1Li})}$, $E_{(\mathrm{TAPQ})}$, $E_{(\mathrm{TAPQ{\bullet}-})}$ and $E_{(\mathrm{Li})}$ denote the total energies of TAPQ−2Li complex, TAPQ−1Li complex, neutral TAPQ, monoanion TAPQ and the Li atom, respectively.

Based on substantial evidences, it can be concluded that four lithium ions can be stored in TAPQ. Therefore, the theoretical capacity is calculated to be 343 mAh g^{-1}. Two lithium ions can be stored in PTQ and DHTAP, the theoretical capacity is 174 mAh g^{-1} and 187 mAh g^{-1}, respectively.

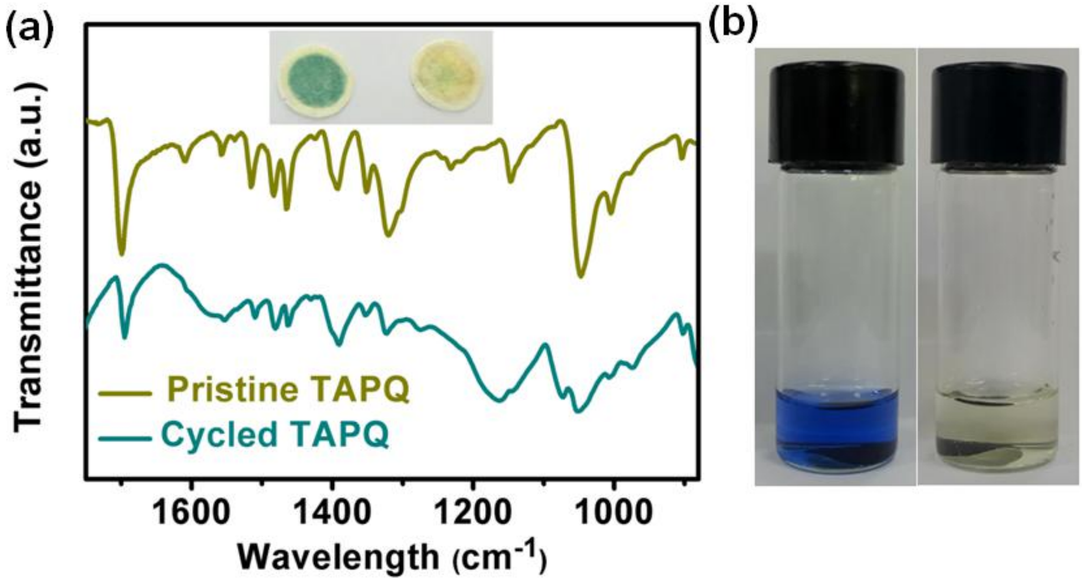

Figure S17. (a) The FTIR spectrum of cycled TAPQ electrode and pristine bulk TAPQ. The insets are the optical photograph of cycled separator. The left one corresponds to the bulk TAPQ electrode. The right one corresponds to the TAPQ/RGO electrode. (b) The solubility of TAPQ (left), TAPQ/RGO (right) in the electrolyte.

Apparently, the cycled separator corresponds to the TAPQ/RGO electrode is slightly become yellow; while the one corresponds to the bulk TAPQ becomes green. This can further testify that composited with RGO is an efficient method to suppress the dissolution of TAPQ.

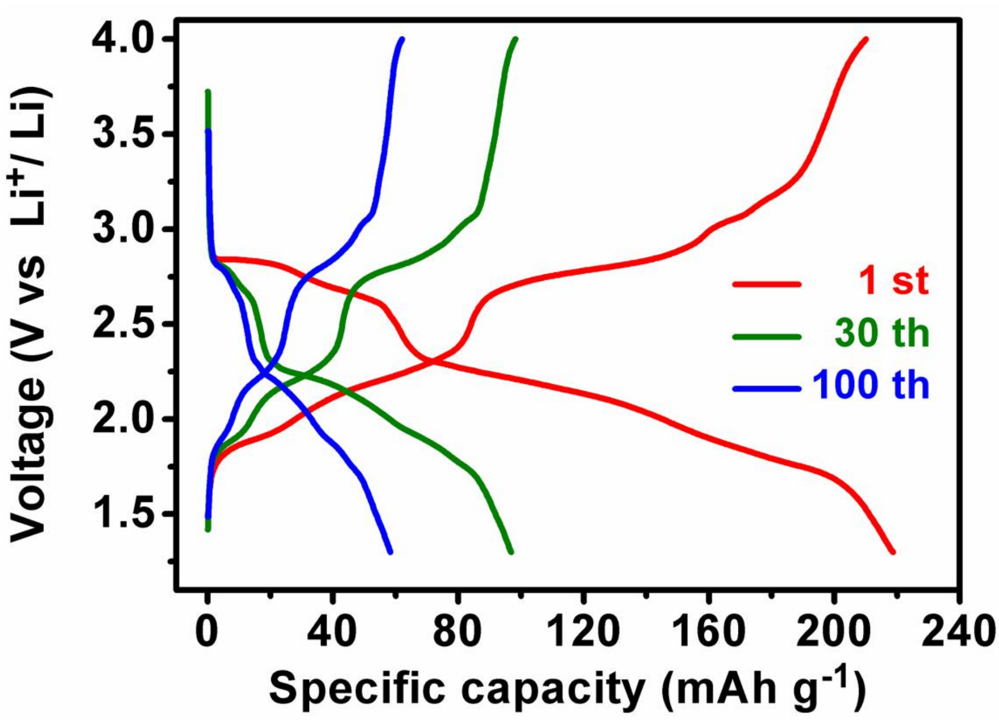

Figure S18. Discharge/charge profiles of TAPQ nanoleaf at current density of 100 mA g^{-1}.

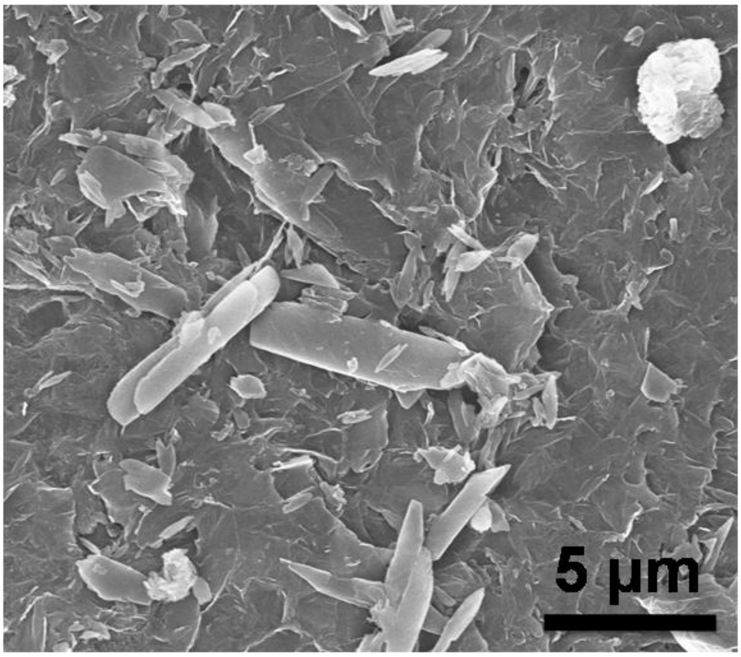

Figure S19. SEM image of TAPQ/RGO-S.

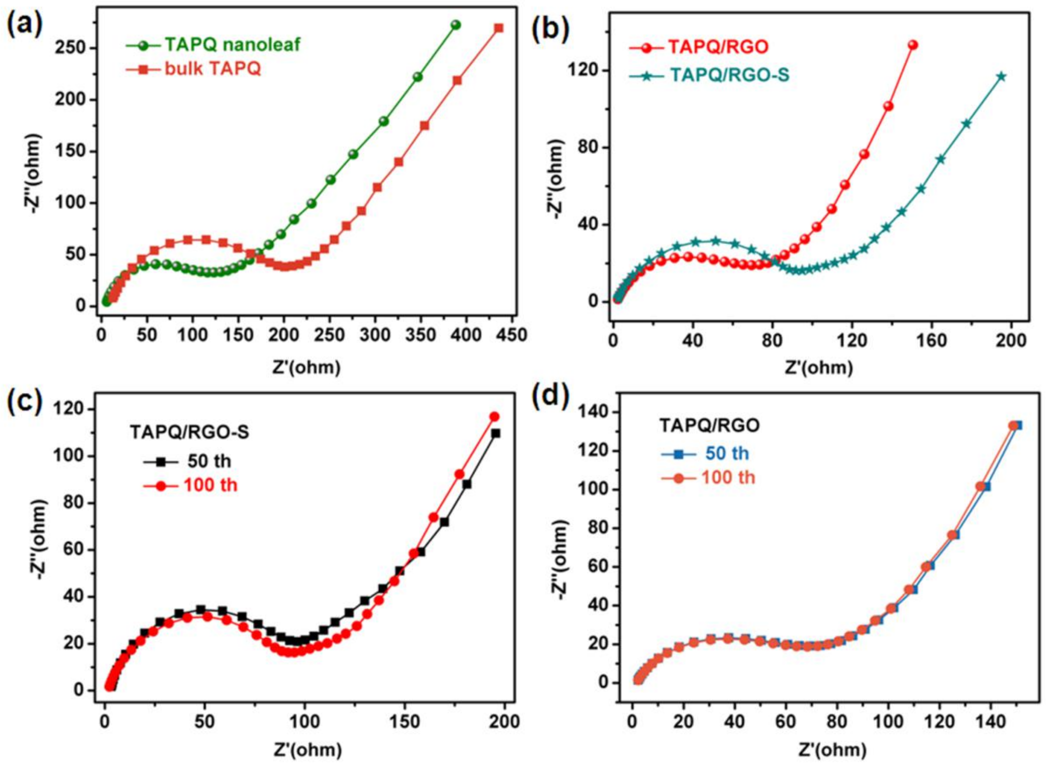

Figure S20. Electrochemical impedance spectroscopy of TAPQ and its composites: (a) TAPQ nanoleaf and bulk TAPQ; (b) TAPQ/RGO and TAPQ/RGO-S after the electrode discharged to 1.3 V at 5th cycle; (c) TAPQ/RGO at 50th and 100th cycle; (d) TAPQ/RGO-S at 50th and 100th cycle.

The changes of electrochemical impedance for TAPQ and its composites indicate that the electrochemical impedance of TAPQ is drastically decreased after composited with RGO. Even after cycling for 100 cycles, the impedance is also remaining stable for TAPQ/RGO, which is better than TAPQ/RGO-S and thus demonstrating the superiority of in-situ composite method.

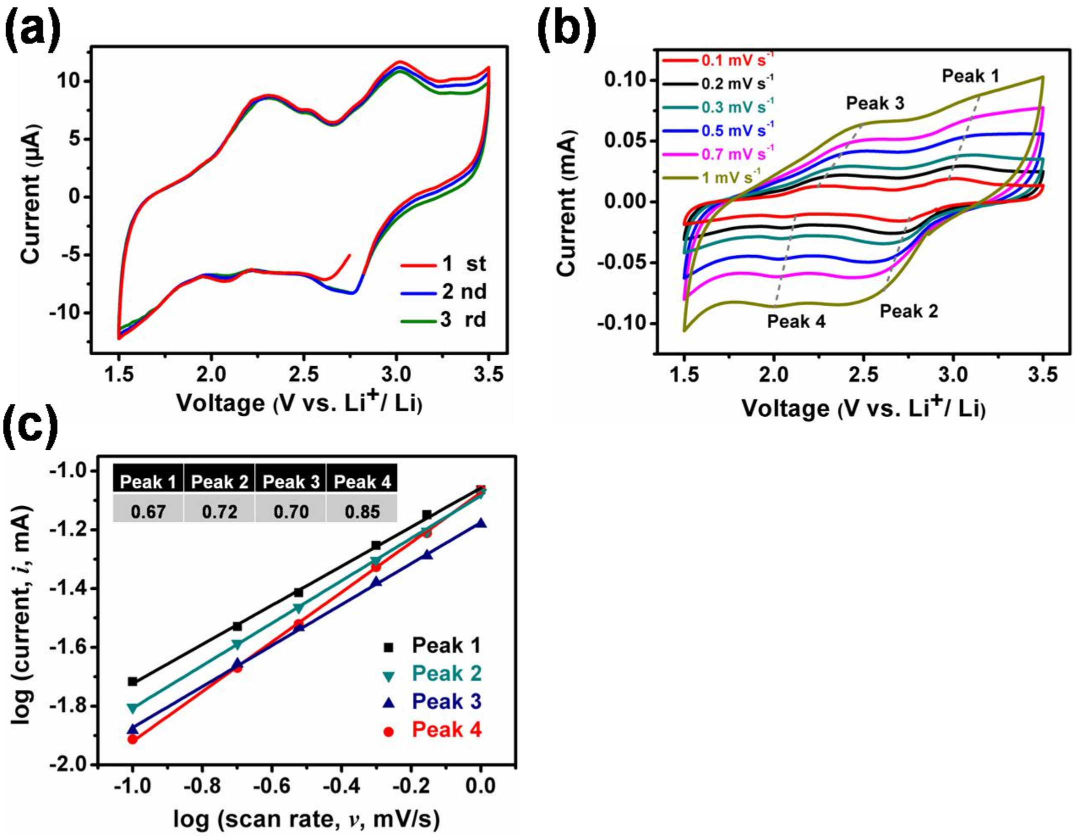

Figure S21: Electrochemical performances of TAPQ/RGO half-cell. (a) CV curves of the initial 3 cycles at 0.1 mV s^{-1}; (b) CV curves of TAPQ/RGO at different scan rates; (c) log(i) versus log(v) plots at different redox states.

As shown in Figure S21a, the redox peaks of TAPQ/RGO in the CV curves were less evident compared with pristine TAPQ. Based upon the discharge/charge profile and CV curves, it can be testified that the intrinsic properties of TAPQ get well maintained even after composited with RGO. As depicted in Figure S21b-21c, CV curves at various scan rates were investigated. With the scan rate increases, the peak current increase is not proportional to the square root of the scan rate. The relationship between peak current (i) and scan rate (v) can be analyzed by the following equations:

$$\log(i)=b\log(v)+\log(a)$$

where i is the peak current, v is the scan rate, a and b are the adjustable parameters. It is obvious that b-values lie between 0.5 and 1, which indicates that the electrochemical reactions of TAPQ/RGO are controlled by the pseudocapacitive behavior and insertion reaction.

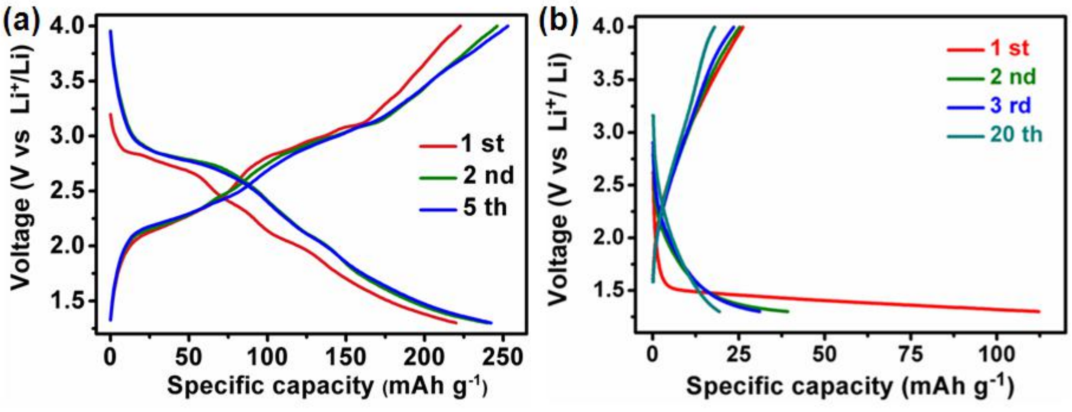

Figure S22. Charge/discharge curves of TAPQ/RGO (a) and RGO (b) at a current density of 50 mA g^{-1} in the voltage range of 1.3-4.0 V.

In order to clarify the capacity contribution, the charge/discharge curves of RGO were measured. Based on the test results, it is evident that the specific capacity of RGO is negligible compared with the high capacity of TAPQ.

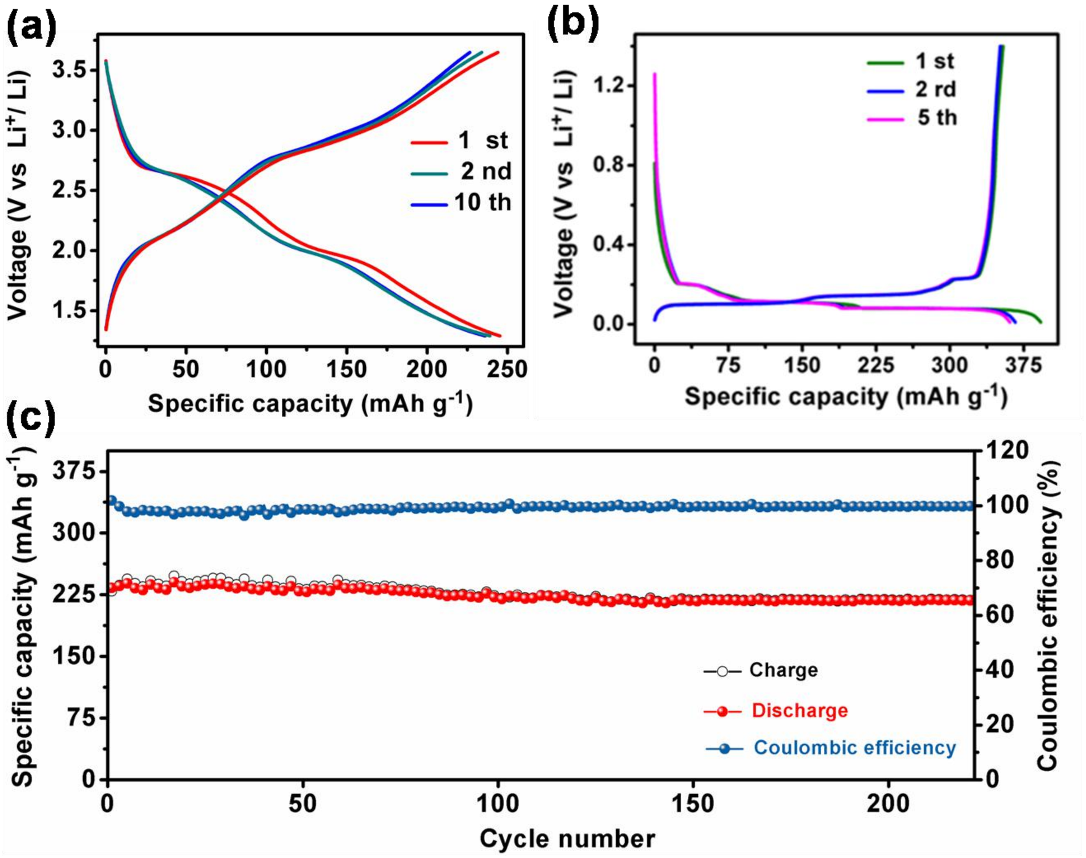

Figure S23. (a) Charge/discharge curves of TAPQ/RGO full cell in the voltage range of 1.29 and 3.65 V at 50 mA g^{-1}; (b) Charge/discharge curves of graphite cycling between 0.01 and 1.4 V at 50 mA g^{-1}; (c) Cycling performance of LIBs full cell by using a TAPQ/RGO cathode and graphite anode at 100 mA g^{-1}.

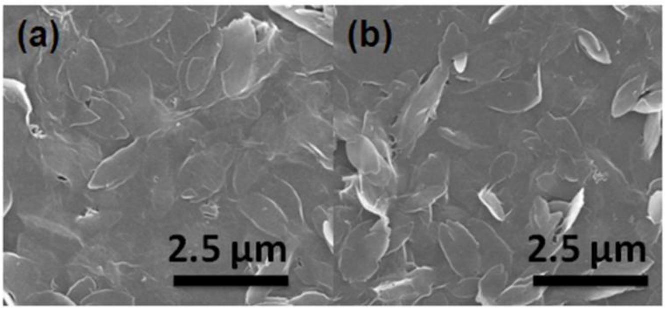

Figure S24. SEM images of TAPQ/RGO electrodes: a) after 50 cycles, b) after 100 cycles.

Comparing the electrode after cycling, the nanoleaf morphology of TAPQ is still maintained and well composited with RGO, and thus demonstrating the superiority of in-situ composite method.

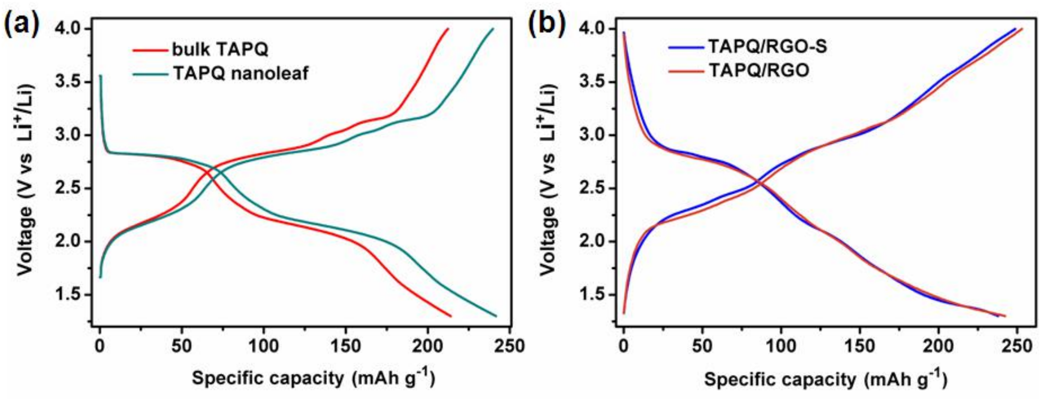

Figure S25. Voltage-Capacity curves of TAPQ and its composites corresponding to Figure 6a and 6b at the current density of 50 mA g^{-1}.

Table S4. Comparison of TAPQ/RGO with those organic/carbon composite electrode materials.

Materials	Capacity ($mAhg^{-1}$)	Capacity retention (Cycles)	Current density ($mA\ g^{-1}$)
This work	193	80% (2400)	500
PI-FLEG(PMDA/EDA)[S5]	108	80% (200)	221
3D-RGO/PI(PMDA/EDA)[S6]	101	82% (150)	221
Juglone/RGO[S7]	212	69.5% (300)	100
AQ/graphene[S8]	120	54%(100)	257
BDTD/graphene[S8]	139	64.95%(100)	243
Carbon coatedporous$Li_2C_8H_4O_4$[S9]	150	67.9% (50)	150
PTCDA/CNT[S10]	90	74%(300)	100
AQ/mesoporous carbon[S11]	97	43.7%(50)	300
H_2bhnq/CMK-3[S12]	203	65.6 (50)	30.9

Supplemental References

[S1] Tang,Q.; Liu, J.; Chan, H. S.; Miao, Q. *Chem. Eur. J* **2009**, *15*, 3965.

[S2] Marcano, D. C.; Kosynkin, D. V.; Berlin, J. M.; Sinitskii, A.; Sun, Z.; Slesarev, A.; Alemany, L. B.; Lu, W.; Tour, J. M. *ACS Nano* **2010**, *4*, 4806.

[S3]Zhu, C.; Guo, S.; Fang, Y.; Dong, S. *ACS Nano* **2010**, *4*, 2429.

[S4] Pandit, V. U.; Arbuj, S. S.; Mulik, U. P.; Kale, B. B. *Environ. Sci. Technol.* **2014**, *48*, 4178.

[S5] Ahmad, A.; Wu, H.; Guo, Y.; Meng, Q.; Meng, Y.; Lu, K.; Liu, L.; Wei, Z. *RSC Adv.* **2016**, *6*, 33287.

[S6] Meng, Y.; Wu, H.; Zhang, Y.; Wei, Z. *J.Mater. Chem.* **2014**, *2*, 10842.

[S7] Wang, H.; Hu, P.; Yang, J.; Gong, G.; Guo, L.; Chen, X. *Adv. Mater.* **2015**, *27*, 2348.

[S8] Liang, Y.; Zhang, P.; Yang, S.; Tao, Z.; Chen, J. *Adv. Energy Mater.* **2013**, *3*, 600.

[S9] Zhang, H.; Deng, Q.; Zhou,A.; Liu, X.; Li, J. *J.Mater. Chem.* **2014**, *2*, 5696.

[S10] Wu, H.; Wang, K.; Meng, Y.; Lu, K.; Wei, Z. *J.Mater. Chem. A* **2013**, *1*, 6366.

[S11] Zhao, L.; Wang, W.-k.; Wang, A.-b.; Yu, Z.-b.; Chen, S.; Yang, Y.-s.*J. Electrochem. Soc.* **2011**, *158*, 991.

[S12] Li, H.; Duan, W.; Zhao, Q.; Cheng, F.; Liang, J.; Chen, J. *Inorg. Chem. Front.* **2014**, *1*, 193.
